# Metabolic and Vascular Effect of the Mediterranean Diet

**DOI:** 10.3390/ijms20194716

**Published:** 2019-09-23

**Authors:** Antonino Tuttolomondo, Irene Simonetta, Mario Daidone, Alba Mogavero, Antonella Ortello, Antonio Pinto

**Affiliations:** U.O.C di Medicina Interna con Stroke Care, Dipartimento di Promozione della Salute, Materno Infantile, Medicina Interna e Specialistica di Eccellenza “G. D’Alessandro” (PROMISE), University of Palermo, Piazza delle Cliniche n.2, 90127 Palermo, Italy; irene.simonetta@live.it (I.S.); mario.daidone@gmail.com (M.D.); albamogavero95@gmail.com (A.M.); antonellaortello@gmail.com (A.O.); antonio.pinto@unipa.it (A.P.)

**Keywords:** mediterranean diet, dietary pattern, cardiovascular risk

## Abstract

Several studies indicated how dietary patterns that were obtained from nutritional cluster analysis can predict disease risk or mortality. Low-grade chronic inflammation represents a background pathogenetic mechanism linking metabolic risk factors to increased risk of chronic degenerative diseases. A Mediterranean diet (MeDi) style has been reported as associated with a lower degree of inflammation biomarkers and with a protective role on cardiovascular and cerebrovascular events. There is heterogeneity in defining the MedDiet, and it can, owing to its complexity, be considered as an exposome with thousands of nutrients and phytochemicals. Recently, it has been reported a novel positive association between baseline plasma ceramide concentrations and cardiovascular events and how adherence to a Mediterranean Diet-style may influence the potential negative relationship between elevated plasma ceramide concentrations and cardiovascular diseases (CVD). Several randomized controlled trials (RCTs) showed the positive effects of the MeDi diet style on several cardiovascular risk factors, such as body mass index, waist circumference, blood lipids, blood pressure, inflammatory markers and adhesion molecules, and diabetes and how these advantages of the MeDi are maintained in comparison of a low-fat diet. Some studies reported a positive effect of adherence to a Mediterranean Diet and heart failure incidence, whereas some recent studies, such as the PREDIMED study, showed that the incidence of major cardiovascular events was lower among those assigned to MeDi supplemented with extra-virgin olive oil or nuts than among those assigned to a reduced-fat diet. New studies are needed to better understand the molecular mechanisms, whereby the MedDiet may exercise its effects. Here, we present recent advances in understanding the molecular basis of MedDiet effects, mainly focusing on cardiovascular diseases, but also discussing other related diseases. We review MedDiet composition and assessment as well as the latest advances in the genomic, epigenomic (DNA methylation, histone modifications, microRNAs, and other emerging regulators), transcriptomic (selected genes and whole transcriptome), and metabolomic and metagenomic aspects of the MedDiet effects (as a whole and for its most typical food components). We also present a review of the clinical effects of this dietary style underlying the biochemical and molecular effects of the Mediterranean diet. Our purpose is to review the main features of the Mediterranean diet in particular its benefits on human health, underling the anti-inflammatory, anti-oxidant and anti-atherosclerotic effects to which new knowledge about epigenetic and gut-microbiota relationship is recently added.

## 1. Introduction: The Rediscovery of Mediterranean Diet

Diet constitutes one of the most relevant environmental factors that influence the development of several chronic age-related diseases. People should learn how to control dietary approach in order to choose those patterns that are associated to promotion of health and prevention of diet-related disease.

Nutrition research has analysed the role of single nutrient with regard of health for several years. Recent knowledge regarding the complex synergistic interactions among nutrients and other food constituents has led to an increased interest in total dietary patterns. The concept of “dietary patterns” has been developed to express a broader and wider analysis of food and nutrient consumption. Among the complex network of interactions existing between genome and epigenetic factors, the application of dietary patterns might thus offer more useful information about the risk to develop some diseases, in particular cardiovascular or cerebrovascular diseases, than single food or nutrient. In this way, several studies indicated how dietary patterns that were obtained from nutritional cluster analysis were able to predict disease risk or mortality [1,2]. Dietary pattern analysis has been pinpointed in the last ten years as a promising and practical approach to evaluate the link between diet and the risk of chronic diseases. Interestingly, dietary pattern research has a developing interest with regard of nutrition policy, particularly as it demonstrates the importance of total diet in health promotion.

Looking at individual nutrients or foods is a partial way that it has been changed by a pattern analysis evaluating the effects of overall diet style. Furthermore, there is growing interest in applying the evaluation of dietary quality indices to study whether adherence to a particular dietary pattern or current dietary guidelines is associated to the risk of disease.

To date, the traditional Mediterranean diet is a well-known and well-studied healthy dietary pattern and many evidences underling how this could represent a central useful tool to control cardiovascular risk factors, such as hypercholesterolemia, diabetes, hypertension [3].

The Seven Countries study, which was a research project that enrolled more than 12,000 men of Finland, Greece, Italy, Japan, Holland, the United States, and Yugoslavia, reported the important role of diet as risk factor for cardiovascular diseases. Of these seven nations, the United States and Finland had the highest animal product consumption, the highest saturated fat intake, the highest cholesterol consumption, and the highest incidence of death from cardiovascular diseases. On the contrary, the Mediterranean countries and Japan showed an opposite trend of cardiovascular event incidence [4].

The Mediterranean diet was reported to present an inverse association with the incidence of coronary artery disease in Southern Europe in comparison with Northern Europe and the United States [5,6]. In the light of these findings, Dr. Ancel Keys discovered (or rediscovered) the Mediterranean diet as a possible healthy diet. In a retrospective article, he described it as ‘*a mainly vegetarian die favouring fruit for dessert*’. 

The Mediterranean diet refers to an eating pattern of the olive growing areas surrounding the Mediterranean Sea. It has been considered to be a “poor man’s” diet, developed over the centuries, as people laboured in order to create sustenance in less hospitable terrain.

This dietary style emphasizes fruits, root vegetables, grains (mostly whole), legumes, nuts, seeds, and olive oil, which represents a hallmark of this dietary pattern, providing a considerable content of monounsaturated fatty acids and reduced intake of saturated fatty acids; moreover, it is characterized by a moderate intake of red wine with meals, a moderate intake of seafood, poultry, dairy products, and low consumption of red meat and sweets. Two typical aspects of a Mediterranean lifestyle include daily physical activity and adequate hydration [3].

In 1995 Ancel Keys affirmed “What is the Mediterranean Diet? One definition might be that it is what the Mediterranean natives eat. But as we know and think of it now, it is a relatively new invention. Tomatoes, potatoes, and beans, for example, came from America long after Christopher Columbus discovered the New World. I noticed that the heart of what was considered the Mediterranean Diet is mainly vegetarian: pasta in many forms, leaves sprinkled with olive oil, all kinds of vegetables in season, and often cheese, all finished off with fruit, and frequently washed with wine” [7].

More recently two researchers fully involved in the study of the cardiovascular effects of Mediterranean diet said that “The term ‘Mediterranean’ diet refers to dietary patterns found in olive-growing areas of the Mediterranean region and described in the 1960s and beyond. There are several variants of the Mediterranean diet, but some common components can be identified: high monounsaturated/saturated fat ratio; ethanol consumption at moderate levels and mainly in the form of wine; high consumption of vegetables, fruits, legumes, and grains; moderate consumption of milk and dairy products, mostly in the form of cheese; and low consumption of meat and meat products” [8].

The Mediterranean diet became part of UNESCO’s intangible cultural heritage in 2010. This distinction highlights the critical role played by this way of eating in the culture and identity of four communities in the Mediterranean basin: Cilento (Italy), Coron (Greece), Soria (Spain) and Chefchaouen (Morocco). In December 2013, other geographic places were added: Agros (Cyprus), Hvar and Brac (Croatia) and Tavira (Portugal). According to UNESCO’s definition, the Mediterranean diet constitutes “*a set of skills, knowledge, rituals, symbols and traditions concerning crops, harvesting, fishing, animal husbandry, conservation, processing, cooking, and particularly the sharing and consumption of food*.”

The present-day lifestyle is characterised by high availability of food and an ever-increasing rate of physical inactivity leading to a situation of apparent psycho-physical well-being, which, however, often does not correspond with the real state of health. The typical eating habits of the Mediterranean populations have been progressively enriched with high-protein foods, saturated fats, and sugars to the point, where intake now exceeds the necessary intake levels. Thus, we are living in an age of “apparent well-being”, where the increase in life expectancy runs parallel to the increase in the risk of diseases such as obesity, metabolic syndrome, cardiovascular disease, and cancer.

Talking about Mediterranean diet not only referred to a dietary pattern, but we also want to stress the role of the “Mediterranean-lifestyle”, which, among several characteristics, includes psychosocial factors, such as interpersonal and family relationships, friendships, enjoying the conviviality of the hosts and other guests; in fact, the new pyramid of the Modern Mediterranean diet, addressed to individuals from 18 to 65 years of age, takes the evolution of society into consideration, highlights the fundamental importance of engaging in physical activity and conviviality, the importance of taking a short nap in the early afternoon, habit that takes different names, depending on the country (siesta, riposo, pennichella), the importance of drinking water, and lays emphasis on the consumption of local seasonal food products.

**Figure 1:**
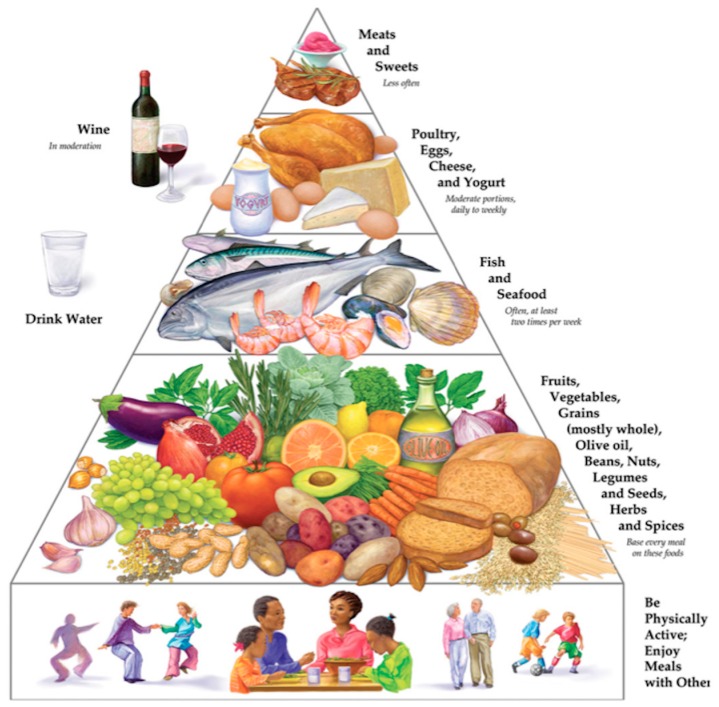
Mediterranean diet pyramid.

It is a Mediterranean diet that has been revised in the light of modernity and well-being, but it also allows for the various cultural and religious traditions and different national identities. The new pyramid is a simple mainframe, which can be adapted to the current needs of the Mediterranean people concerning all local variants of the Mediterranean diet (Figure 1). 

**Figure 2:**
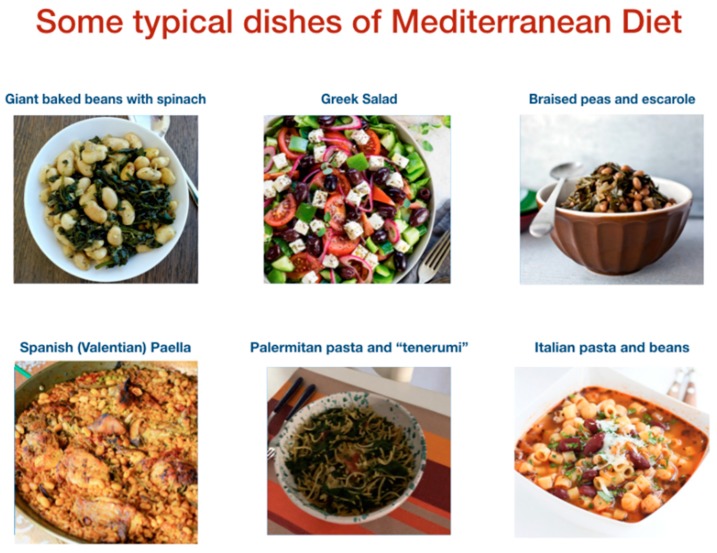
Some typical dishes of Mediterranean diet.

The Mediterranean diet is not a homogenous and exclusive model among the 16 countries that are situated around the Mediterranean Sea (Figure 2). Despite fruit, vegetables, cereals, olive oil are typical food of the Mediterranean diet each country has its own dietary habits influenced by socio-cultural, religious, and economic factors. For example, pork that is one of the most frequent types of meat consumed in European countries of the Mediterranean basin [9], is rejected in Muslim countries on religious grounds. Typical Mediterranean fermented beverages as wine are excluded in Muslim countries, which have a higher consumption of tea and fruit juices. Another difference has been observed regarding virgin olive oil consumption that is low in countries of the Middle East and Northern Africa in comparison to Spain, Italy, or Greece. 

Mediterranean populations, such as Greece, Italy, and the Mediterranean coastal regions of France and Spain, adopt a food model that contains the typical meals of the Mediterranean dietary pattern, however they have preserved their eating habits over the years. These differences are especially evident when comparing the characteristics of the main meals in the four Mediterranean countries. 

Pasta, which is one of the main foods of the Mediterranean diet pattern, is more widespread in Italy, where it is always present in the main meals. Another important food is bread, which is often served after pasta in Italy. Greeks often serve a bowl with olive oil together with bread.

Mixed dishes of rice, vegetables, meat, poultry, or fish are combinations that distinguish Spain from France, where these foods are typically served one at a time and below. Instead of them, Greek population prefers stews vegetables and meat without the addition of rice or potatoes, usually served separately. 

Eggs are widespread in the main Spanish meals, a tradition that distinguishes them from the other Mediterranean populations. In France, in most cases, cuisines, such as pâté or other processed sauces, are served at the beginning of the meal. 

Greek and Spanish populations both consume fewer cheeses than the other two Mediterranean populations. However, the amount of feta that is present in the green salad, a typically Greek dish that also includes olives and olive oil, makes this dish a single dish. 

In the Mediterranean populations, the salads are very-common dishes, which are quite simple because they are not very rich in ingredients and frequently seasoned with olive oil and vinegar. A typical habit of Provence and Spain is to put them on the table at the beginning and then to serve them before the main course, unlike other Mediterranean countries. A salad that is common from the Strait of Gibraltar to Eastern Greece, it provides any variety of lettuce seasoned with a little salt, a couple of drops of vinegar, and abundant olive oil [10]. 

Even though the geographical position of Portugal, which is not in the Mediterranean basin, Mediterranean diet is an integral part of cultural heritage of Portuguese population; this is prevalently due to ancestral influences from the Mediterranean neighbours and from migrants. The Portuguese diet is also influenced by cultural factors, socio-economic context, genetic background, and increased availability of fish due to its wide Atlantic coast. However, several factors have contributed to withdrawing the Portuguese population from traditional dietary habits, for example European Union agriculture policies and financial capacity to afford foods of quality reported by Portuguese population. According to recent ecological studies, Portugal has experienced a relevant decrease in Mediterranean diet adherence over the last decades. A consequence of this trend is the inexorable increase of obesity, as it was observed in women of childbearing age and pregnant women [11]. 

North African countries bordering the Mediterranean Sea share dietary habits that are typical of European Mediterranean countries. However, these customs are influenced by social and economic changes. In the Moroccan population it has been described a higher consumption of bottled water, infusions, and juices [12].

Benhammou et al. compared the dietary customs of three Mediterranean populations (Spain, Morocco, and Palestine) with different cultures and lifestyles, but are influenced by the same geographic context, The adherence to Mediterranean diet was evaluated through MD Serving Score (MDSS). After the analysis of the questionnaires, which were administered to all participants, they described a moderate adherence to the Mediterranean dietary pattern in all populations. In comparison to the Palestine population, MDSS-assessed adherence to the Mediterranean diet was 6.36-fold higher in the Spanish population and 3.88-fold higher in the Moroccan population.

The Spanish diet appeared to be closer to Mediterranean recommendations than the diet of Morocco or Palestine.

Common components were fruit, vegetables, olive oil, while fish and alcohol did not appear in the Lebanese dietary pattern.

In Spain, it was described a high intake in proteins and lipids and low in carbohydrates; on the other hand, carbohydrate intake was higher and protein intake lower in Palestine than in Spain or Morocco [13]. Belahsen et al. also showed a high- carbohydrate intake in Egypt, Jordan, Libya, Tunisia, and Algeria, with reduced fat intake in comparison to the MD recommendation (Food and Agriculture Organization, FAO, 2005) [14].

In the Palestinian population, it was reported a reduced calcium consumption, which may be a consequence of their low dairy product consumption. Moreover, Palestinian participants had a very low iodine intake in comparison to Spanish and Moroccan populations. Additionally, FAO reports have pointed out iodine deficiency in Palestine and Morocco: it likely is responsible a more limited access to iodized salt in these countries (FAO, 2005; FAO, 2011).

The findings of this study could be explained by the low intake of vegetables, fish, and dairy products by the Palestinians. In conclusion, the diet of the Spanish participants is closer to MD recommendations in comparison to the diets of the Moroccans and Palestinians, despite the three groups belonging to the Mediterranean basin [15]. Currently, a debate exists about the adherence to the Mediterranean diet in modern Greece and Cyprus: their diet is the same traditional Mediterranean diet or these countries have moved toward a more Westernised diet? According to the results of recent ecological studies, Greece shows the most relevant drop in the Mediterranean dietary pattern over the last decades.

A systematic review by Kyriacou A. et al. examined the current evidences regarding the adherence of the Greek and Cypriot population to the Mediterranean diet.

All of the studies reported consistent moderate adherence to the Mediterranean diet. These results were independently observed, whether the original Mediterranean diet score and cut-off points were applied or whether results were converted to percentages and tertiles were used as the cut-off points. 

This systematic review suggests that adherence to the Mediterranean diet has decreased since the 1950s and 1960s, when it was high by definition. This observation could be related to a recent changed interest from single nutrients to whole diets in the last decade [16]. For example, Dedoussis et al. described, after an analysis of elderly subjects from five different countries, that Greece had a statistically significant lower Mdiet score when compared with Italy, a higher one than Poland, but no appreciable difference from Germany and France [17]. Van Diepen et al. compared the Mdiet score of Greek and Dutch university students. The results of this study showed an unexpected more increased statistically significant adherence in favour of the non-Mediterranean origin Dutch students [18].

In the light of these recent studies, the observed change in the diet of Greek population seems to reflect a transition from the traditional Mediterranean diet to a more Westernized diet, thus partially explaining the worsening in the health of this country. Recently, it has been observed a relentless increase in the incidence of diseases as diabetes, obesity cardiovascular disease, some cancers, and dyslipidaemia [19]. Other contributing non-diet related factors are the high prevalence of smoking and reduced physical activity observed in the Greek population [20]. 

Given the well-known beneficial healthy actions of the Mediterranean diet, public health and education measures should be implemented to reverse this trend [21]. 

With regard to Croatia, results from a recent study suggest that differences in dietary intake and food choices are becoming less noticeable in rural versus urban parts and are less geographically dependent with “westernized diet” taking place instead of a “traditional diet”. This study, which was conducted in school aged children, reported that the prevalence of obesity among Croatian children was high and unrelated to an urban/rural setting. It is necessary to coordinate the role of family together with educational institutions and health professionals in order to interrupt this raising negative trend [22].

In respect of the well-established impact of good dietary habits on the prevention of cardiovascular diseases and other chronic diseases, health policies should focus on adherence to a healthy diet, as Mediterranean diet, supporting traditional dietary patterns in a period of intense commercial pressures.

In the light of the above-mentioned considerations, we would review the main effects of the Mediterranean diet, especially on inflammation, atherosclerosis, and cardiovascular diseases.

## 2. Biological Effects of Mediterranean Diet 

Over the years, several studies have been carried out on the effects of the Mediterranean Diet, some of which have been relevant in order to know the mechanisms that could explain the potentially beneficial role of the Mediterranean Diet for the prevention of cardiovascular disease (CVD). In this setting, the biomolecular mechanisms that seem to promote long-term health effects of the Mediterranean diet are the following: lipid-lowering effect; protection from oxidative stress, inflammation and platelet aggregation; inhibition of nutrient sensitization pathways through specific restriction of amino acids and production of intestinal metabolites that are mediated by the microbiota (increase in genome stability). 

### 2.1. Role of Genome

The knowledge of the gene-diet interaction might allow to better clarifying the mechanisms through which a modifiable factor as diet influences gene expression. Accumulating data are emerging about the different responses of individuals with different genotypes to diet; the study of genetic predisposition might provide a tool to detect those subjects who may benefit from intervention on dietary patterns.

The first studied gene in order to identify the genetic variants associated to a high susceptibility to cardiovascular disease was the gene encoding ACE (angiotensin converting enzyme). This study reported that homozygous individuals for a deletion in the chosen gene presented a superior risk of myocardial infarction. APOE gene is another example of studying genetic variants that are related to susceptibility to cardiovascular disease, and still the CEPT (cholesterol ester transfer protein) gene was studied in animal models and human studies demonstrating its role in reverse cholesterol transport, thus influencing proatherogenic and antiatherogenic processes [23,24].

The genome wide approach intends to search some associations between many SNPs and diseases. 

Single nucleotide polymorphism (SNPs) are common genetic variants that are detected through methods of genotypization in the DNA sequence. An important goal of GWAS (genome wide association study) is to understand and investigate the relationship between single nucleotide polymorphisms and phenotypes [25]. 

In the field of the studies regarding Mediterranean diet the results of the PREDIMED trial highlight the strict influence of the dietary components on genetic predisposition towards cardiovascular risk. In this regard, Carella and colleagues studied the rs7903146 polymorphism in the transcription factor 7-like2 gene; in particular, they reported a relationship between the genetic predisposition to present hypercholesterolemia, hypertriglyceridemia, augmented fasting glucose, and stroke incidence in those subjects that are homozygous for the T-allele of the above-mentioned polymorphism. Their results suggest that the adherence to the Mediterranean diet might attenuate the effect of this genetic predisposition [26]. 

Ortega Azurim et al. found an association between the rs3812316 polymorphism at the MLXIPL gene (MLX interacting protein like) with reduced circulating triglycerides levels and they reported that the Mediterranean diet modifies the triglycerides (TG) concentrations. The protective effects of the Mediterranean diet against cardiovascular risk were mainly evidenced in subject who presented higher adherence to this dietary approach. In particular this study pointed out that the reduction of cardiovascular risk, which was related to the adherence to Mediterranean diet, was especially accentuated in carriers of the G-allele at MLXIPL locus [27].

Telomere length is modernly considered a marker of biological age and its involvement in gene-diet interaction has recently been described. Other studies, in particular observational and ex vivo works, have reported that a long-term adherence to Mediterranean diet seems to be related to higher telomere length, activity. For example, Garcia Calzon et al. reported longer telomeres in women joining Mediterranean dietary approach, but not in males.

Further analysis suggests that some gene variants might play a role in modulating telomere length following Mediterranean diet approach; for instance, it has been considered Pro/Ala polymorphisms of the peroxisome proliferator activated receptor γ2. It seemed that the carriers of Ala variant had longer telomeres. Other observations were improvement in obesity parameters, dietary inflammatory markers; these modifications probably lead to a slowed down process of shortening of telomere [28,29,30,31]. 

A group of 521 subjects from the PREDIMED study has undergone genetic analysis in order to evaluate the expression of Ala allele in rs1801282 of PPARγ2 gene (peroxisome proliferator activated receptor γ2). After a five-year follow up, it was observed that subjects following a constant strict adherence to the Mediterranean Diet showed benefits because of their longer telomeres. The detection of an interaction between gene and diets could represent a further element that highlights the importance of personalized dietary approaches in order to favour healthy aging [32].

The circadian locomotor output cycles kaput gene (CLOCK gene) has been recently studied by Garcia Ros and colleagues in order to give data regarding the hypothesis that the habitual adherence to a healthy dietary pattern as Mediterranean diet seem positively influence glucose metabolism; this effect appeared to be mediated by interaction with the rs1801260 SNP in the CLOCK gene. Another study by Lopez Guimera et al. investigated the interaction of CLOCK 3111T/C polymorphism in the response of weight loss program; the authors found that this polymorphism seemed to have effects on emotional eating behaviour thus influencing weight loss. In the light of these results, genetic predisposition and higher exposure to Mediterranean diet impact on several traits, such as lipid profile, glucose metabolism, emotional eating behaviour, and telomere length [33,34].

### 2.2. Role of Epigenome 

In the context of gene-diet interaction, it is placed epigenetics, a fascinating area of research that in the future might allow for understanding the molecular basis of this interaction, and thus to predicting the risk of development disease and help to define prevention strategies and therapeutic approaches. Epigenetic studies the changes in the genome might cause alterations in gene expression without determining modification in the DNA sequence. Epigenetic mechanisms include noncoding RNA function, DNA methylation, and histone modifications [8]. Diet may produce epigenome changes, for example some nutrients constitute methyl donating components, as methionine, folic acid, B vitamins, choline. The first impact of nutrients and their eventual imbalance presents effects since foetal life; in fact, studies conducted on animal models but also human studies suggest that an altered supply of nutrients during gestational periods, infancy is associated with a major risk of mortality for cardiovascular diseases. Both excess and deficiencies of some dietary components might induce epigenetic alterations that thus result inherited and influence phenotype. In this connection, previous studies [35,36] reported a significant evidence of a relationship of maternal nutrient deficiency and epigenetic perturbation leading to a higher cardiovascular risk and an augmented risk of development chronic disease. An animal model pointed out that female offspring (APOE +/-) presented a high susceptibility to form neointima after exposition to maternal hypercholesterolemia (APOE-/-). This observation was different to that reported in same offspring that was exposed to wild type mothers. Fetal programming and the exposition to specific dietary pattern thus seem to modulate epigenomic processes and histone methylation. Another study in similarity with previous observations reported that APOE-/- mice, which were more susceptible to atherosclerosis, showed some alterations of DNA methylation that favoured the development of atherosclerosis [37,38]. 

Accumulating data point out the intricate relationship existing between diet and specific food component and epigenetic mechanisms. Epigenomic wide association studies are an interesting novel field of research that might allow for defining the key role of epigenetics and its changes in the context of the development of disease in response to diet and environmental factors. Since early phases of life, nutritional intake may induce DNA modifications through epigenetic changes, thus influencing susceptibility to develop disease [39,40].

Several studies have reported that microRNAs constitute, among other functions, regulators of numerous processes as apoptosis, lipid metabolism, cell differentiation, and glucose metabolism. microRNA act inducing the silencing of their target genes inhibiting mRNA [41]. 

Richardson et al. observed that the minor allele of the single nucleotide polymorphism rs 13702 in the 3′UTR of LPC8 (lipoprotein lipase) gene determined the disruption of the recognition site for micro RNA410. This effect resulted in gain of function, and finally in reducing the plasma levels of triglycerides. This study reports the molecular basis of some cardiovascular effects and that these molecular mechanisms seem to involve miRNAs and different regulation pathways.

### 2.3. Role of Nutrigenomics

Nutrigenomics or nutritional genomics is a science studying mechanisms by which diet and its nutrients interact with genes, therefore it represents an approach for investigating the different responses of subjects after the same dietary interventions. These differences are studied in several fields: genetics, transcriptome, metabolome, and epigenetics [42].

The mechanisms through which nutrients could modulate gene expression could be a direct binding to nuclear receptors or act indirectly modulating epigenetic effects. Microarrays and real time reverse transcription PCR are modern techniques by which to study gene expression modification [43].

Nutrigenomic studies have reported the protective effects of Mediterranean diet on vascular inflammation, thrombosis, and foam cell formation through a modulation of the expression of pro-atherosclerotic genes, also, the content of antioxidants represents one of the elements that is responsible of the benefits of Mediterranean diet.

Konstatinidou’s study suggests that both Mediterranean diet and olive oil induce gene expression changes if constantly taken [44]. 

Vazquez and colleagues analysed the metabolomics pattern of non-diabetic subjects participating to PREDIMED study. They assigned the participants to two groups: low fat diet and Mediterranean diet; they studied their urinary metabolomic profile with proton nuclear magnetic resonance. The main hallmarks detected in the second group were 3 hydroxybutyrate, citrate, cisaconitate that are related to carbohydrates metabolism, proline, glycine, *N*-acetylglutamine branched chain amino acids that are related to the metabolism of amino acids, creatine, oleic, and subenic acids, derived from lipids, and finally microbial cometabolites as p-cresol phenyl aceteylglutamine. Moreover, they studied the metabolomics pattern that was related to walnut intake; this was studied through high-performance liquid chromatography–quadrupole-time of flight mass spectrometry (HPLC-Q TOF-MS). 18 metabolites were identified in that group undergoing dietary pattern enriched with walnut: they include allagitannin, derived microbial compounds, metabolites of the tryptophan/serotonin pathway, of fatty acid metabolism [45].

Gonzalez Guardia et al. studied the metabolomic profile of a group of elderly subjects that were assigned to Mediterranean diet supplemented with Coenzyme Q10 (CoQ10). They reported higher hippurate urine concentrations after four weeks of exposure to Mediterranean diet+ CoQ10; on the other hand, in the group following a saturated fat diet, they observed increased levels of phenylacetylglycine. This study suggests that long term exposure to a Mediterranean dietary approach rich in CoQ10 is associated to beneficial effects on healthy aging and prevent mechanisms of chronic oxidative stress.

The above-mentioned studies and other literature evidences show how nutrigenomics trough the knowledge of the processes driving gene-diet interaction, but also of metabolic processes, could finally provide individualized and accurate recommendations to prevent the development of several diseases.

### 2.4. Role of Microbiota

Mediterranean foods also have an impact on gut microbiota that, during the last few years, has been identified as a possible risk factor of cardiovascular disease representing a potential therapeutic target; so, microbiome could represent a possible intermediate of the effects of the Mediterranean diet of modulation of cardiovascular risk factors [46,47]. 

Microbioma is the study of host enteric microbial ecology; metabolomics studies the role of low molecular weight metabolites in biological samples. New insights come from the deeping of these fields of research, reporting the systemic effects of gut microbiota, which are direct through a direct interaction between microbes and host and indirect through the production of de novo metabolites or that derive from diet. It was already known the role of gut microbioma in nutrient metabolism, enteric immunity, in the production of micronutrients.

Limited studies have investigated how a complete dietary pattern might affect the metabolomic profile; instead, numerous studies have examined the effects of single specific dietary components on metabolome (low molecular weight metabolites). In this stimulating field, it is possible to place the study of the effects of Mediterranean diet on cardiovascular outcomes through its effects on gut microbiota [48]. 

Over recent years, increasing evidence suggests that microbiome mediates the effects of Mediterranean diet on metabolic mechanisms involved in the development of chronic diseases, in fact it is clear that food intake and some specific components modify enteric microbiota inducing changes that may be responsible of some effects exerted by Mediterranean diet.

The discovery of new biomarkers is one of the latest goals of nutritional metabolomics to define a personalized diet as possible preventive tool and for managing diseases.

Some studies have delineated the metabolomics profiles in several dietary patterns as low-fat diet, low glycaemic index diet, the New Nordic diet, very low carbohydrates diets, on the other hands, currently there is little evidence regarding the metabolomics profiles in Mediterranean diet and their association with the state of health.

Gonzalez-Guardia et al. conducted a crossover study in which they examined some biomarkers in four groups of subjects following an isocaloric dietary pattern: the Mediterranean diet, the Mediterranean diet supplemented with CoQ (Coenzyme Q10), the Western diet that is rich in saturated fat, a low fat, high carbohydrate diet rich in n-3 polyunsaturated fat. In the second group, they found higher urinary concentrations of hippurate and in the Western diet group they reported higher levels of phenylacetylglycine in female participants. The greater levels of hippurate showed a positive correlation with a circulating concentration of CoQ and ββ carotene and a negative correlation with the genetic expression of superoxide dismutase 1, thioredoxin, transcription factor Nrf2, the gp91phox subunit of nicotinamide adenine dinucleotide phosphate oxidase gene [49]. 

Kakkoura et al. investigated the effects of the interplay of some polymorphisms with the Mediterranean diet on the serum levels of fourteen metabolites that were selected for their role in influencing the expression of gene encoding enzymatic proteins that are modulated by specific known polymorphisms. A higher adherence to Mediterranean approach was associated with an increased MTHF [50]. Another cross-sectional study reported urinary microbial metabolites in particular phenylacetylglutamine, p-cresol, 4 hydroxyphenylacetate were linked to a greater adherence to Mediterranean diet [51]. 

As regards the Mediterranean diet, several studies have recently evaluated the metabolites in the PREDIMED trial; metabolites that were assessed include ceramides, tryptophan, acylcarnitine, glutamine, choline pathway metabolites. In particular, an increased cardiovascular risk has been documented in the presence of elevated levels of choline pathway metabolites, ceramides, glutamine, short chain triacylglycerol, monoacylglicerol, and acylcarnitines [52,53,54,55,56,57]. Meanwhile, a reduced risk of cardiovascular disease has been reported in presence of greater cholesterol esters, polyunsaturated phosphatidylcholines, and a major glutamine/glutamate ratio. The Mediterranean diet exerts a protective role in the field of cardiovascular disease counteracting the harmful effects of such metabolites, this association appear more relevant when baseline metabolites are evaluated. 

However, a large variability in the metabolomic profiles of Mediterranean pattern emerges from these studies, the difference and sometimes the conflicting results might derive from different biological samples, the analytical methods, the study design, and the Mediterranean diet composition. In the future, it is desirable to abolish the above-mentioned diversities standardizing methods.

Many nutrients, particularly protein and insoluble fiber, as evidenced by numerous metagenomic data, have a strong impact on gut microbiota function, structure, and production of metabolic substances that are able in modulation of the immune response and in the activation of many metabolic and inflammatory pathways [56,57]. In fact, the restricted content of cholin and I-carnitine (substances abundant in red meat, eggs, and cheese), as compared to other dietary regimen, leads to a reduced cardiovascular risk: high levels of TMAO (trimethylamine *N*-oxide), product of the metabolism of choline and I-carnitine, determine an increase of vascular inflammation and a direct prothrombotic effect by the promotion of platelet hyper-responsiveness to multiple agonist both in humans and rodents, and these are probably associated to the pathogenesis of obesity and type 2 diabetes. High levels of TMAO are associated to an increased risk of developing cardiovascular disease in both humans and mice, regardless of risk factor [58,59].

Bacteroidetes (in particular, Bacteroides acidifaciens) have an important role in gut microbiota through the production of high levels of short-chain fatty acids, including acetate, propionate, or butyrate, in fact many experimental animal data suggest that their high levels can discontinue the progress of several inflammatory, autoimmune and allergic disease [60]. Some authors think that some of these useful effects could be mediated by the binding to specific G-protein coupled receptors, placed on enteroendocrine and immune cells. The importance of gut microbiota is also underlined by a recent randomized clinical trial, which has demonstrated the reshape of gut microbiota in a population of obese adhering to Mediterranean diet for two years, which leads to an increase of Bacteroides, Prevotella, and Faecalibacterium genera, and most importantly of the Roseburia and Ruminococcus genera and Parabacteroides distasonis and Faecalibacterium prausnitzii bacterial species, known for their saccharolytic activity and the ability to convert carbohydrates in short-chain fatty acids [61]. Accumulating evidences suggest that poor diet in “microbiota-accessible carbohydrates” can result in the extinction of specific bacterial lineages, which leads to the modification of maturation process and the function of immune system, which causes an increased risk of developing metabolic, inflammatory, allergic, and autoimmune disease [62,63], differently, the long term adherence to healthier plant-rich diets, through the reprogramming of human gut microbial functions, can play an important role in promoting health and longevity [64,65]. 

### 2.5. Effects on Inflammation Markers

Inflammation is a crucial element in the pathogenesis of endothelial dysfunction as well as atherosclerosis [66]. Strictly inflammatory elements, such as cytokines and adhesion molecules expressed at the level of endothelial cells, as well as white blood cells, play an important role from the early stages of atherosclerosis, as they promote the recall of inflammatory cells that are present in the circulation, thus contributing to the development of atheromatous plaque [67].

In this context, several clinical and epidemiological studies have been conducted suggesting how the beneficial role of the Mediterranean diet in the control of the main risk factors for the development of arteriosclerosis could be in explained part through an anti-inflammatory effect on the endothelial side, as shown by an improvement in endothelial function that is associated with a reduction in the status of inflammation following the adoption of a Mediterranean dietary model [68,69,70,71]. 

Equally important results were reported by Estruch, who showed, in a sub-study of the PREDIMED trial [72], the effects of the Mediterranean diet enriched with virgin olive-oil (VOO) or nuts on 106 individuals with a high cardiovascular risk [69]. The results suggested that, in contrast to what was evident after the adoption of a low-fat diet, Mediterranean diet (MD) with VOO supplement could play its role by changing the plasma levels of protein C and Soluble vascular cellular adhesion molecule-1 (sVCAM-1), as shown by the results that were obtained. In addition, this author showed that MD enriched with VOO or nuts were both associated with a significant reduction, after three months of observation, of the plasma levels of IL-6 as well as the expression of CD49d, an adhesion molecule that is expressed by circulating inflammatory cells in peripheral blood, which is essential for their recruitment and levels of the CD40 ligand.

Other experimental and clinical studies have found a reduction in cellular expression of vascular cell adhesion molecule-1 (VCAM-1) and intercellular adhesion molecule-1 (ICAM-1) associated with a decrease in E-selectin expression by endothelial cells as a result of olive oil consumption [73]; similar results were shown for plasma Soluble intercellular adhesion molecule-1 (sICAM-1), E-selectin, IL-6, and finally CRP [68,70].

As suggested by Estruch, these results support the hypothesis that the benefits obtained from the Mediterranean diet enriched with VOO or walnuts are partly associated with its anti-inflammatory effects, as shown by the reduction of levels of inflammatory cellular and circulating molecules that have always played a key role in the development of one of the most important risk factors, such as atherosclerosis. For such motivations, lifestyle changes that include the adoption of a dietary model, such as MD, can play a key role in CVD prevention.

## 3. Effects of Mediterranean Diet on Cardiovascular Risk Factors

It is now known, thanks to numerous findings from several randomized controlled trials [74,75], the Mediterranean style exerts positive effects on most of cardiovascular risk factors, such as body mass index, waist circumference, blood lipids, blood pressure, inflammatory markers and adhesion molecules, and diabetes; moreover, accumulating data report that these advantages of the Mediterranean diet are maintained also in comparison of a low-fat diet. We would like to briefly present some of the latest scientific evidence about the effects of Mediterranean diet on diabetes, hypertension, lipid profile, and thus on atherosclerosis.

### 3.1. Mediterranean Diet and Diabetes

Several randomized clinical studies have shown that a Mediterranean-type diet, which is made up of different kind of vegetable, results in a significant reduction in body weight, unlike high-glycemic index foods belonging to a Western diet. In fact, a 5-month randomized trial showed that women who practiced a Mediterranean diet achieved a weight reduction of about 4 kg. These patients also reported a reduction in blood glucose, peptide C values, and fasting serum testosterone levels, insulin reduction after glucose tolerance test, and a marked increase in serum levels of Insulin-like growth factor-binding protein 1–2 (IGFBP-1, IGFBP-2) and growth hormone binding protein. This study, besides an improvement in body weight and in metabolism, also showed a reduction in the risk of developing breast cancer, although these data should be verified in further studies [76]. In relation to this, it has been hypothesized that the Mediterranean dietary regime that is involved the production of short chain fatty acids with a consequent increase in intestinal hormones (GLP-1 and PYY), which inhibit gastric emptying inducing satiety [77]. 

It has also been hypothesized that the intake of foods with a low glycemic index and a low content of branched-chain amino acids, as well as an increased intake of monounsaturated acids, might lead to a reduction in insulin resistance and consequent hyperinsulinemia [78,79].

In this regard, a study that was conducted on rodents showed that reduced methionine intake led to an improvement in the glucose profile and a reduction in the risk of obesity and hepatic steatosis. It is also determined an increase in serum levels of FGF2 and adiponectin, with a reduction of plasma levels of IGF-1, leptin and T4. The Mediterranean diet does not only include low intake of methionine, but also of other essential amino acids (such as tryptophan, valine, leucine, and isoleucine), which would seem to modulate the insulin sensitivity.

In recent years, several studies have suggested that dietary models with high MUFA content have positive effects on diabetes [80]. In this context, several studies have been conducted, some of which on subjects that were predisposed to insulin resistance [81,82] and others on healthy individuals [70,83], which suggested that dietary Mono-Unsaturated Fatty Acids (MUFA) consumption was associated with an optimization of both glycemic control and insulin sensitivity, contrary to food with saturated fatty acids (SFA), which can negatively affect these variables [84], particularly in cellular elements, such as smooth muscle cells [85].

For example, Lopez et al. conducted a study on 14 healthy men with an average age of 27 + /− 7 and BMI (in kg/m^2^) of 23.9 +/− 1.9 in 2008, which had a single-blind, randomized, within-subject crossover design; the results of this study suggested that progressive replacement of SFA by MUFA had an effect on the function of pancreas B cells and on insulin sensitivity, resulting in progressive improvement of the same in the postprandial period [86].

Prior to the study conducted by Lopez et al., Vessby et al. in the KANWU study, a 2001 study that was based on 162 healthy individuals who followed a dietary model rich in SFA or MUFA for three months showed how the high-SFA diet was associated with worsening insulin sensitivity [83]. In this study, it was also shown, ensuring a daily consumption of fat that was equal to 37% of the total energy, an 8.8% increase in sensitivity to this pancreatic hormone following a diet rich in MUFA instead of SFA, which instead caused a 12.5% reduction in this hormone.

However, other studies have assessed whether high MUFA diets had different effects compared to dietary models with a high Carbohydrate (CHO) portion, focusing attention on a possible improvement in sensitivity to insulin to prevent the development of resistance to insulin, and therefore type 2 Mellitus Diabetes through specific changes in dietary habits.

For example, Garg conducted a meta-analysis in 1998 based on ten different studies comparing the nutritional effects of different dietary models, in particular high-CHO and high-MUFA diets, in patients with diabetes. A comparison of these two different dietary approaches showed that MUFA consumption is associated with optimization of lipoprotein profile and glycemic control [87], results that were subsequently confirmed by Ros in 2003 which reassessed the relationship between the adoption of a dietary model with a high MUFA content and a glycemic control level in patients with type 2 Mellitus Diabetes [88].

Paniagua et al., with a prospective study including eleven offspring of patients with obesity and diabetes divided into three groups, each following three different dietary plans each lasting 28 days, showed how, when compared to consuming a diet that was rich in carbohydrates or saturated fats, the MUFA-enriched diet has positive effects on insulin-resistant subjects through an improvement of Homeostasis model assessment for insulin resistance (HOMA-IR), a substitute for insulin sensitivity and proinsulin levels. Another important result that emerged from this study was the increase in the amount of HDL-C and GLP-1 associated with the lowering of glucose and insulin plasma concentrations in the postprandial phase after a breakfast that was enriched with olive oil, contrary to what has been shown following a high carbohydrate diet [82].

Other studies confirmed a positive relationship between MUFA consumption and metabolic control when compared to high CHO and SFA diets. Due et al. conducted a randomized controlled trial based on 20 men and 26 women, randomly divided into three groups with different diets, in order to compare and evaluate the effect of diets that are enriched with fats and carbohydrates on carbohydrate metabolism. This study revealed that, after six months, the MUFA-rich diet had positive metabolic effects through an improvement in variables, such as fasting glycaemia, and insulin, reduced by 3% and 9.4%, respectively, and insulin sensitivity, as suggested by the reduction of the homeostasis model assessment of insulin resistance score of 12.1%. In contrast, the other two dietary approaches have been associated with an increase in these three variables [81]. In addition, several studies have been conducted over the years to compare these different food approaches in order to assess their nutritional effects on healthy subjects, as shown by the results of these studies, there is no significant divergence between the plasma levels of the main metabolic control markers in subjects who have adopted three dietary plans with different macronutrient compositions [89,90] (See Table 1).

### 3.2. Mediterranean Diet and Hypertension

Hypertension is the most common cardiovascular disease, which is defined as the persistence of systolic and/or diastolic blood pressure (BP) values ≥ 140 and 90 mmHg, respectively. Elevated blood pressure causes pathological changes at the vascular and cardiac level. For this reason, hypertension is one of the main risk factors for cardiovascular diseases and renal failure [93].

According to current guidelines for the treatment of hypertension that were published by the European Society of Cardiology (ESC) and the European Society of Hypertension (ESH), based on meta-analysis, reviews of randomised controlled studies, observational studies, and systematic reviews of studies in adults, the development of hypertension can be prevented, in combination with a reduction in cardiovascular risk, by implementing lifestyle changes [94]. In particular, salt restriction and moderation of alcohol consumption in association with the high consumption of vegetables and fruits have proven to be effective strategies for reducing blood pressure. Therefore, one of the optimal dietary patterns for hypertension control is one that includes a high consumption of vegetables, legumes, fruits, low-fat dairy products, cereals, fish, and olive oil associated with low consumption of red meat and foods containing saturated fatty acids [95,96]. The traditional Mediterranean diet (TMD), which includes most of these foods, has been correlated with blood pressure (BP) reduction. The large-scale, randomized and controlled clinical trials, such as the PREvención study with DIeta MEDITERRÁNEA (PREDIMED), showed how the administration of TMD enriched with extra virgin olive oil (EVOO) or nuts to patients at high risk of cardiovascular disease had effects on the primary prevention of these diseases. In this study, lower BP values were shown in patients who received TMD + EVOO or TMD + nuts when compared to the control group [97].

Nissensohn et al. conducted a systematic review and meta-analysis to examine the benefits of MD, which was compared with a low-fat diet, on both systolic and diastolic blood pressure [98]. In this study, six controlled randomised trials were included and they evaluated, in more than 7000 subjects, those that were the effects of a Mediterranean diet pattern after an intervention period of at least one year. For example, Toledo et al., in a randomised controlled study in 2013 based on 7447 subjects with high cardiovascular risk that has been included in this meta-analysis, they showed, after four years of follow-up, a significant improvement in diastolic blood pressure in subjects who had been assigned to the two groups promoting the Mediterranean diet that was enriched with extra virgin olive oil or nuts compared to those who had been allocated in the control group, who had been instructed to follow a low-fat diet [99]. In view of the results that were obtained with this meta-analysis, Nissensohn et al. showed a significant reduction in both systolic and diastolic blood pressure in subjects with normal blood pressure or mild hypertension, after consumption of a Mediterranean diet. In particular, the researchers pointed out that the effects of the Mediterranean diet are more significant for systolic blood pressure, although the statistical impact of the meta-analysis was influenced by the small number of studies that were included.

One of the major organs that participates in the regulation of pressure homeostasis is the endothelium. Endothelial cells are able to synthesize molecules, such as nitric oxide (NO) and endothelin 1 (ET-1), substances with vasodilatory action and vasoconstrictor, respectively [100,101]. A lack of balance between the production of NO and that of ET-1 induces endothelial dysfunction [102]. Several studies have shown that the reduction in NO levels and the increase in ET-1 are both involved in the pathogenesis and development of hypertension, in combination with modifications of the expression of their receptors [103,104].

Storniolo et al., in a recent trial of 2015 that was based on ninety non-smokers women divided in three groups with different dietary plans, they showed that the TMD that was enriched with EVOO or nuts partly reduced BP in hypertensive women inducing changes in the plasma levels of endothelial-derived factors, such as NO and ET-1, and regulating the expression of the receptor gene for the ET-1 [105].

Another interesting result of this trial was a significant increase of NO metabolites concentration in TMD + EVOO group, however the serum concentration of ET-1 was significantly lower in the TMD + nuts group; therefore, the results that were obtained in this study suggested that the NO levels were modulated by the components of extra virgin olive oil while the levels of ET-1 were controlled by the nutrients contained in the nuts.

In recent years, many studies have shown that an important contribution to the pathogenesis of hypertension is given by the effective reduction of NO bioavailability.

Storniolo et al. showed that TMD + EVOO is associated with both an increase in eNOS levels, with a consequent increase in NO release, and a reduction in the expression of the gene that codes for the Caveolin-2 protein, according to the proposal by Konstantinidou et al. [106], who hypothesized that the TMD that was supplemented with VOO (virgin olive-oil) had positive effects by regulating the expression of genes that were related to cardiovascular risk. Caveolin-2 is a crucial element of the inner surface of small invaginations of the plasma membrane, called caveolae, and it is involved in various cellular processes, including the inhibition of eNOS. Therefore, olive-oil components could partly reduce the BP by negatively regulating the expression of caveoline, with a consequent increase in the bioavailability of NO [107].

In the last years, a significant role on reducing the cardiovascular and cerebrovascular risk profile has been given to the strict interaction gene-environment with a particular focus on dietary influences on genes expression [108].

In 2013, Castaner et al. conducted a study on peripheral blood mononuclear cells (PBMNCs) by using whole transcriptome microarray analysis on a subsample of 34 subject of the PREDIMED trial with the aim to evaluate if the beneficial effects of TMD were referred to nutrigenomics influences [109]. In the particular case of arterial hypertension, the authors identified seven different molecular pathways that were related to the disease, such as hypoxia, nitric oxide, eNOS, P2Y purigenic, cardiac hypertrophic signalling, aldosterone, and renin-angiotensin. The effects of these pathways could be referred to JUN gene downregulation that was observed in patients treated with a polyphenol-rich olive oil, according to the proposal of Camargo et al. [110], which have hypothesized that the benefits that are associated with a polyphenol-rich could be determined by the effect on AP-1 activation, which lies of heterodimer c-JUNcFOS. In particular, Camargo et al., showed how the anti-inflammatory effects of the polyphenol-rich olive oil components could be mediated by the modulation of the expression of certain genes that took part in NF-kB/AP-1 signalling pathways. When also considering that NF-kB was able to modulate the expression of iNOS [111], which was an enzymatic isoform expressed after stimulation by inflammatory cytokines, the polyphenols contained in olive oil could exert their protective effect both by inhibiting these inflammatory pathways and by improving endothelial function.

Moreno-Luna et al., in a double-blind randomized, crossover dietary intervention study of 2012, after four months long follow up, they showed that polyphenols, which are important components of EVOO, were able to reduce BP in young women in stage 1 of essential hypertension promoting an increase in the bioavailability of NO [112]. In this trial it has been showed the consumption of Polyphenol-rich olive oil was associated with an increasing of plasma levels of nitrites/nitrates and with a reduction of ADMA, an endogenous substance that inhibits NO synthesis [113].

Moreover, Moreno-Luna et al., in their study, they revealed a reduction in the amounts of oxidative stress and inflammation mediators, such as Oxidized low-density lipoprotein (ox-LDL) and C reactive protein (CRP) levels, in association with the consumption of Polyphenol-rich olive oil.

The ox-LDL are able to activate the renin-angiotensin system, which in turn activates Lectin-like oxidized low-density lipoprotein receptor-1 (LOX-1) that is a receptor for ox-LDL trough angiotensin II type I receptor, thereby exacerbating oxidative stress [114]. CRP is known for being responsible for endothelial dysfunction and the way in which it exercises its role is to negatively alter the stability and the uncoupling of NO synthase mRNA, therefore CRP represents a ligand of Lectin-like oxidized LDL receptor-1 (LOX-1), taking part both in inflammation and oxidative stress, which are strongly related to the development and maintenance of hypertension [115,116].

Entotelin-1 is another molecule that performs a key role on endothelial dysfunction and so on development of hypertension.

As noted above, Storniolo et al. [105] also documented a reduction in the serum levels of ET-1 in subjects that submit a Mediterranean diet regimen implemented with nuts. In association with the reduction of ET-1 levels, the results of this study suggested that TMD is able to determine the downregulation of receptors that mediate the contractile effect of ET-1, such as ETAR (endothelin A receptor) and ETBR (endothelin B receptor), which are implicated in the pathogenesis of hypertension. In this way, the benefit that is associated with nut consumption on BP could be mediated, in part, through reduced expression of ETAR and ETBR on muscle and endothelial cells, respectively, and the depletion of ET-1 levels.

One of the peculiarities of the Mediterranean diet is the consumption of olive oil, which is characterized by the high ratio between the content of monounsaturated and saturated fatty acids. Psaltopoulou et al. [117] conducted a general population study, including 20343 volunteers from Greece aged between 20 and 86, who had previously been enrolled to participate in the EPIC study, a study that had been conducted in 22 research centres in 10 different European countries. The aim of the EPIC study was to assess the contribution of factors, such as dietary, environmental, biological, or lifestyle-related factors to the pathogenesis of chronic diseases, such as cancer [118,119]. Psaltopoulous et al. showed that the adoption of the Mediterranean diet was inversely related to blood pressure values. In particular, another interesting result of this general population study was the significant association between olive consumption and changes in both the systemic and diastolic values of BP, contrary to another important component of the Mediterranean diet, such as cereals, currently positively related to BP [120,121].

In accordance with these results, other studies have shown how the long-term consumption of high amounts of virgin olive oil (VOO), thus characterized by high layers of monounsaturated fatty acids (MUFA) with particular attention to oleic acid (OA) (70–80%), which is present in triglycerides, promotes the lowering of BP, thus preventing the development of chronic high tensive levels [74,122,123,124] OA (cis-18: 1n-9) can influence a key element of BP control, such as the adrenergic receptor, as demonstrated by recent studies [125]; it has been suggested that this effect could be due to changes induced by fatty acid, at the level of the structure of cell membranes. The lipid component of the membrane can also be modified by 2-hydroxyoleic acid, which represents a structural analogue of the same OA; these molecules are sufficient for regulating the same intracellular signal transduction pathways [126,127]. In confirmation of what has been described, Teres et al., in a study that was conducted in 2008, emphasized that the antihypertensive properties of olive oil could partly be due to the high content of OA. In fact, previous studies have mainly attributed to the minor components of olive oil, such as alpha-tocopherol, polyphenols, and other phenolic compounds, the protective effect of one of the fundamental and characteristic foods of MD [128,129,130,131].

Teli et al., when comparing the effects of VOO, triolein (TO), the preponderant constituent of VOO (characterized by a TG with three molecules of OA) and OA on BP, they showed that OA, both free and esterified, was in able to positively modify the amount of cis-MUFA in the membranes, influencing the conformation of the cell membrane (2,3 di 8), contrary to elaidic acid (trans 18: 1n-9 isomer of OA) and stearic (18: 0), a result that suggests that the structure of the molecule is important to have hypotensive properties. By increasing the distance between the polar heads of membrane lipids [132,133] by increasing the concentration of cis-MUFA, OA and structural analogues or precursors define what are the site, the degree of activity, and the concentration of components of important signal transduction pathways, with particular attention to the 2A/D-adrenoreceptor/G protein/adenylyl cyclase-cAMP/PKA system [125]. In addition to favouring the expression of cAMP and PKA, whose activation is positively associated with vasodilation, this study showed that the consumption of VOO, TO, and OA induced a reduction in the concentration of proteins with vasoconstrictor action, which include the three isoforms of alpha subunit of G proteins (Galfa i2, Galfa i3, Galfa q/11) and PLCbeta, with a consequent reduction of inositol phosphate, diacylglycerol, and calcium [134]. Another important beneficial effect that was supported by the results of the present study is that OA negatively influences the concentration of these second messengers through a direct mechanism, inhibiting the synthesis of diacylglycerol and inositol (1,4,5) triphosphate and interfering with the cascade of calcium-activated intracellular signalling [135]. With these data, Teres et al. emphasized that the consumption of OA is significantly associated with an improvement in vasodilation systems together with a reduction in the vasoconstriction pathways (See Table 2).

### 3.3. Mediterranean Diet and Lipid Levels

Many studies, which were carried out both in the past and in the recent present, have proved how an adherence to the Mediterranean diet is strongly related to a reduction in blood pressure values and to a beneficial alteration in glycemic and lipid profile [74,117].

Atherosclerosis represents the primary cause of cardiovascular diseases [136], which is due to the fact that cholesterol-rich lipoproteins and apolipoprotein B become oxidized in the arterial subendothelial matrix and this process determines the production of pro-inflammatory molecules which activate the endothelium itself. This process is also mediated by monocytes, which are recruited from the bloodstream. Once reaching the site, these cells become foamy cells after having phagocytized the oxidized LDL, contributing to the increase in the size of the plaque and to the chronicization of the inflammatory process [137,138]. The therapeutic strategies that were chosen in the treatment of these pathologies range from the medical approach to the lifestyle approach [138]. On this basis, various substances, contained in many products of Mediterranean diet, have been analysed, as they seem to cause healthy and protective effects in cardiovascular field.

The extra virgin olive oil, the omega-6 and omega-3 fatty acids taken with the consumption of shelled fruit and plant sterols represent the main source of fatty acids taken with the Mediterranean diet [139,140].

The increased consumption of water-soluble fibres (contained in fruit and beans) has shown, in numerous randomized controlled studies, a significant reduction in plasma LDL cholesterol concentrations (each gram involves a reduction of about 1.12 mg/L of LDL cholesterol) [141,142].

Another induced effect is represented by a reduction in the reabsorption of cholesterol and bile acids in the intestine, with consequent absorption in the liver.

A significant amount of α-tocopherol, carotenoids, oleuropein, and phytosterols are contained in 100 g of extra virgin olive oil. Furthermore, olechantal is contained in the newly pressed extra virgin olive oil; this phytosterol seems to inhibit COX activity, such as ibuprofen [129]. The dose that was contained in 50 g of extra virgin olive oil does not seem to have powerful anti-inflammatory effects, but it seems to guarantee protection against platelet aggregation.

A recent study by Allaire et al. [143] showed that the intake of DHA (docosahexaenoic acid), compared to EPA (eicosapentaenoic acid), determined a significant reduction of CRP, TNF alpha, IL 6, and triglycerides, with an increase in the concentrations of adiponectin and HDL cholesterol, in subjects with high cardiovascular risk.

Carotenoids (a-carotene, b-carotene, lycopene, lutein, fucoxanthin, canthaxanthin, zeaxanthin, b-criptoxanthin, capsorubin, and astaxanthin), which are mainly found in fruits and vegetables [144], have antioxidant effects and they are involved in the regulation of the immune system and in the transduction of messages within the cell [145,146]. Carotenoids are also implicated in the atherosclerotic process that causes a slow of progression of atherosclerotic plaque [147,148]. A study that was conducted by Cheng et al. [136] showed that the intake of tomatoes (rich in carotenoids) contributed to a reduction in plasma LDL cholesterol concentrations.

Mozos et al., in their review, showed that, among carotenoids, lycopene, which is a bright red carotenoid hydrocarbon found in tomatoes and other red fruits and vegetables, such as red carrots, watermelons, gac melons, and papayas, is one of the most relevant because of its beneficial effect, such as antiatherosclerotic, antioxidant, anti-inflammatory, antihypertensive, antiplatelet, anti-apoptotic, and protective endothelial effects, the ability to improve the metabolic profile, and reduce arterial stiffness [149]. However future studies are needed to better understand the mechanisms through which lycopene exerts its favourable actions on pathogenesis of cardiovascular disease.

Phytosterols cannot be produced directly by humans, so it is necessary to assume them with diet. Foods that are rich in phytosterols are vegetable oils, some cereals, but above all nuts [150,151]. Gylling H et al. [152] showed that the intake of 2 g/day of phytosterols contributed to reducing LDL cholesterol by about 10%.

Nuts, fruits, and vegetables also contain another antioxidant substance: coenzyme Q [153]. This coenzyme is present at the mitochondrial level and shares some elements with the biosynthetic pathway of cholesterol, such as mevalonate. Treatment with statins results in a reduction of this coenzyme and this appears to be associated with an increased risk of developing cardiovascular disease, therefore individuals receiving statin treatment might experience a reduction in CoQ10 levels [154,155].

Zhang et al. [156] showed, in a meta-analysis based on 15 studies, how the supplementation of this coenzyme to medical therapy resulted in a better glycemic and lipid profile control in patients with type 2 diabetes mellitus.

Ambring et al. [157] conducted a study that evaluated endothelial function in healthy patients with high lipid levels who practiced a Mediterranean diet. The results showed that the concentrations of the various lipid components were more diminished in individuals who had practiced a Mediterranean diet when compared to those with a Swedish diet.

Hernáez et al. [158] demonstrated that the Mediterranean diet supplemented with extra virgin olive oil, in people with high cardiovascular risk, promoted the five main functions fulfilled by HDL: cholesterol efflux and metabolism, anti-oxidant, anti-inflammatory and vasodilatory effect.

The basic function of HDL is to guarantee the efflux of cholesterol from peripheral to liver cells, where it is released for metabolization [159]; This action is inversely related to the risk of developing cardiovascular diseases [159] and major coronary events [160]. Once cholesterol is internalized, HDLs esterify it through the LCAT enzyme (Lecithin–cholesterol acyltransferase) [161]. This activity is assessed while using the HDL cholesterol esterification index, which allows for evaluating the LCAT activity. The authors of this study showed that a Mediterranean dietary pattern supplemented with extra virgin olive oil increased the concentration of esterified cholesterol. This positive effect is probably due to the antioxidant properties contained in the healthy foods of the Mediterranean diet, which protect the LCAT enzyme from oxidation, guaranteeing its normal functionality [162].

Another function that is performed by HDL is the antioxidant activity, which, contrasting LDL oxidation, prevents the formation of atherosclerotic plaques) [163]. This effect is determined by the action that was carried out by the enzyme PON1 (Serum paraoxonase/arylesterase) [164]. This enzyme appears to be a predictive biomarker of cardiac events [165], although its activity is still being studied in humans [166]. The study that was developed by Álvaro Hernáez et al. showed that the Mediterranean diet determined an increase in the arylesterase activity performed by PON1. In addition, the Mediterranean diet that was supplemented with extra virgin olive oil promotes the induction of nitric oxide synthase at the endothelial level by HDL [158].

Endothelial homeostasis is also guaranteed by the efflux of ABCG1-dependent cholesterol that is determined by HDL rich in sphingosine-1-phosphate [167].

Sphingosine represents a marker of degradation of ceramides, which are a product of sphingolipid metabolism. A recent study [168] evidenced how the consumption of walnuts results in an increase in sphingosine concentrations without causing changes in dihydroceramide concentrations (de novo ceramides synthesis marker). These data support the hypothesis that us based on the concept that the reduction of the total ceramide levels is caused by increasing their catabolism, due to their more intense activity. The results of this study showed that the consumption of walnuts determined a reduction of the small LDL levels, particles with a pro atherogenic power greater than the large LDL [169]. This study also showed an increase in plasma concentrations of large HDL, whose levels were inversely related to the risk of developing cardiovascular diseases [170]. In this regard, the FDA has established that the consumption of a certain amount of walnuts (about 42.5 g/day) contributes to the reduction of the risk of developing cardiovascular diseases [171]. Moreover, walnuts are very rich in polyunsaturated fatty acids (PUFA), primarily alpha-linolenic acid (ALA), an omega-3 fatty acid with anti-atherogenic effects [172].

A recent study [173] was designed randomizing patients with a low-moderate cardiovascular profile in two arms: the first, which was assigned a Mediterranean dietary regimen and the second a low-calorie dietary regimen. The results that were obtained did not show significant changes in terms of BMI and blood pressure, except for a reduction in the concentration of IL-17 induced by the Mediterranean diet.

Furthermore, a study that was conducted on people who had followed a Mediterranean diet [174] and subjects with a low-fat dietary regimen revealed how the first diet determined a reduction of hepatic fat content (HFC) and visceral adipose tissue (VAT). This reduction in HFC was greater in patients with Mediterranean diet as well as healthy effects. The results of this study evidence how the reduction of hepatic fat content could play an important role on determining the beneficial effect by a Mediterranean dietary regimen, even more consistently than visceral adipose tissue.

Another study [175] evaluated the relationship between a major cardiovascular event (acute myocardial infarction) and other cardiovascular risk factors in southern European patients undergoing a Mediterranean dietary regime. These results showed that the intake of carbohydrates and saturated fatty acids was directly related with BMI increase in men but not in women, whose BMI changes were associated to protein intake instead. They also evidenced that, in men, the plasma HDL-C concentration was inversely related to BMI.

A sub-study [176] of the Attica study that was conducted on overweight and obese people showed that increased insulin sensitivity and a better dietary profile were achieved in those subjects with greater adherence to a Mediterranean diet.

#### Effects on Plasma Ceramides

Ceramides are precursors of complex sphingolipids. Some cellular and animal models reported the pathogenetic role of the accumulation of ceramides in the activation of several signalling targets that impair normal cellular functions that also involve insulin action. Furthermore, this issue also seems to be related to an increased de novo ceramide biosynthesis in response to cellular stress stimuli, such as the exposure to saturated free fatty acids (FFAs) [169].

Ceramide and its metabolites have been reported as an intermediate point between excessive feeding and metabolic abnormalities that cause cardiometabolic disease risk, such as insulin resistance and low-grade inflammation [177,178].

However, to date, available evidence about ceramides and health outcomes mostly comes from in vitro experiments and animal studies, and it is mainly based on intermediate outcomes of cardiovascular risk.

Few studies have prospectively investigated the association between ceramides and the incidence of cardiovascular and cerebrovascular events.

A recent study [179] analysed the relationship between ceramides and major adverse cardiovascular events (MACEs) among apparently healthy individuals. Havulinna et al. quantified four circulating ceramides, Cer(d18:1/16:0), Cer(d18:1/18:0), Cer(d18:1/24:0), and Cer (d18:1/24:1), in 8101 serum samples by a targeted liquid chromatography-tandem mass spectrometry assay. Authors observed the strongest association with incident MACE and the highest unadjusted hazard ratio of 1.31 (95% confidence interval, 1.21–1.41) for Cer(d18:1/18:0), which remained significant at 1.21 (95% confidence interval, 1.11–1.33) after Framingham risk factor adjustments. The conclusion of this study was that distinct serum ceramides were associated with the risk of incident of major adverse cardiovascular events in apparently healthy individuals. These results suggest the role of ceramides in cardiovascular pathobiology and suggest their role as possible new biomarkers of MACE risk.

Another recent study [180] measured six previously identified high-risk plasma ceramide molecules [Cer(d18:1/16:0), Cer(d18:1/18:0), Cer(d18:1/20:0), Cer(d18:1/22:0), Cer(d18:1/24:0), and Cer(d18:1/24:1)] in 167 consecutive patients with established or suspected Coronary Artery Disease (CAD) who underwent either exercise or dypiridamole myocardial perfusion scintigraphy (MPS) for various clinical indications. After adjustment for age, sex, smoking, dyslipidaemia, hypertension, diabetes, prior history of CAD, left ventricular ejection fraction, and type of stress, testing were independently associated with the presence of inducible myocardial ischemia. Thus, this study showed how plasma ceramides are positive and independent predictors of stress-induced myocardial perfusion defects in patients with established or suspected CAD referred for clinically indicated myocardial perfusion scintigraphy.

Recently, Laaksonen et al. reported an unclear relationship between plasma ceramides CV events underlying the role of two ceramides as the strongest predictor of CV events among patients with coronary artery disease.

Wang et al. [54] evaluated participants from the PREDIMED trial (Prevención con Dieta Mediterránea), including 230 incident cases of CVD and 787 randomly selected participants at baseline (including 37 overlapping cases) followed for ≤7.4 years. The participants were randomized to a Mediterranean diet supplemented with extra virgin olive oil, a Mediterranean diet supplemented with nuts, or a control diet. In this study, the ceramide score, which was calculated as a weighted sum of concentrations of four ceramides, was associated with a 2.18-fold higher risk of CVD across extreme quartiles. Participants with a higher ceramide score and assigned to either of the 2 active intervention arms of the trial showed similar CVD risk to those with a lower ceramide score, whereas participants with a higher ceramide score and assigned to the control arm presented significantly higher CVD risk. These findings suggest a positive association between the baseline plasma ceramide levels and cardiovascular events and they report how adherence to a Mediterranean diet style may influence the potential negative relationship between elevated plasma ceramide concentrations and CVD.

### 3.4. Effects of Mediterranean on Atherosclerosis

Cardiovascular diseases, which include coronary artery disease, cerebrovascular diseases, peripheral artery diseases, and heart failure, constitute the main cause of mortality worldwide. WHO (World heart organization) estimates that cardiovascular disease will cause the death of 23.6 million people by the year 2030. Atherosclerosis is the foremost cause of cardiovascular disease, especially coronary artery disease and stroke; its risk factors include metabolic factors, genetic predisposition, environmental factors, and behavioural habits [8].

Current knowledge highlights the key preventive role of the Mediterranean diet in the context of cardiovascular disease; in this regard, in the last decades, several studies and some randomized dietary intervention trials as the Lyon diet heart study have reported the beneficial effects of Mediterranean style on the secondary prevention of the cardiovascular diseases. The PREDIMED study constitutes the first randomized controlled and large-scale intervention trial that shows the significant impact of the Mediterranean diet as primary prevention tool against cardiovascular events, such as stroke, atrial fibrillation, peripheral vascular disease, and myocardial infarction [181,182]. Subsequently, the PREDIMED trial reported that the adherence to Mediterranean diet enriched with EVOO or nuts was associated with a reduction of 30% of cardiovascular events as compared with those reported in a control group following a low-fat diet; the population of PREDIMED was high risk primary prevention one [183].

Atherosclerosis represents the cause of almost 90% of cases of acute coronary syndrome, 60% of strokes, the majority of case of chronic heart failure, peripheral arterial disease, and vascular dementia.

Hypercholesterolemia constitutes one of the fundamental risk factors of atherosclerosis. Numerous studies have reported that decreased intake of saturated fat is associated with lower levels of plasma cholesterol and lesser incidence of coronary heart disease; this relationship has been observed, in particular, when saturated fat is substituted with polyunsaturated and monounsaturated fat. The substitution of 5% of energy intake from saturated fats from the same quantity of energy from polyunsaturated fats, monounsaturated fats, or carbohydrates from whole grains, is correlated to 25%, 15%, and 9% reduced risk of coronaropathy, respectively; instead, substituting saturated fats with carbohydrates from refined grains is linked to raised risk of coronary heart disease. These observations have been confirmed by various randomized controlled clinical trials that have shown a reduction of approximately 30% of cardiovascular disease incidence when dietary saturated fats are replaced with vegetable polyunsaturated fats. This percentage reduction is comparable to that induced by statin therapy. A better and lasting cardiovascular benefit could be obtained if plasmatic cholesterol would be maintained lower throughout life, promoting the prevention of atherosclerosis.

The traditional Mediterranean diet is characterized by a low consumption of meat, milk, and butter, so the intake of saturated fats is almost 8% of energy, on the other hand, a higher intake of total fat derives from extra virgin olive oil, various types of nuts, seeds, the germ of whole grains, constituting 25% to 35% of calories. Prospective studies have reported that the consumption of five servings of nuts per week leads to a 40% to 60% reduction of coronary heart disease events. A great contribution in reducing LDL-cholesterol and cardiovascular risk is given by nuts, especially almonds, hazelnuts, walnuts, and pine nuts, which are a rich source of omega 6 and omega-3 fatty acids. A high intake of water soluble fiber, of which a traditional Mediterranean diet is rich and which are found in beans and fruits, is associated, as reported in some randomized controlled studies, with a relevant plasma cholesterol lowering; according to some hypothesis water soluble fiber causes a decreased reabsorption of cholesterol and bile acids in the small intestine, leading to increased LDL uptake by the liver [142].

The traditional Mediterranean diet includes very low amount of partially hydrogenated trans fatty acids that are involved in the pathogenesis of coronaropathy. Replacing calories from mono or polyunsaturated fat with trans fatty acids is associated with increased LDL cholesterol levels, apolipoprotein B, triglycerides, lipoprotein, and reduced plasma levels of high-density lipoprotein cholesterol and apolipoprotein A 1.

The first step in the management of hypertension, which is one on the main risk factors of atherosclerosis, requires, among first steps, to follow an healthy diet, as the Mediterranean diet that includes foods rich in vegetables, fruits is a resource of phytochemicals: it is known that these latter show an inverse association to hypercholesterolemia and hypertension [3].

A recent study by Shafqat and colleagues investigated the effects of Mediterranean dietary pattern on a population of healthy US women with long term follow up, in particular they analysed 40 biomarkers that are involved in glucose metabolism, lipid metabolism, inflammation, branched chain amino acids, and apolipoproteins. Moreover, the authors aimed to characterize novel factors that could contribute to the relationship between Mediterranean diet and risk reduction in cardiovascular events; the results of this study support the role of higher Mediterranean diet intake as preventive tool of cardiovascular disease, even in non-Mediterranean diet populations. The positive effects of Mediterranean diet that were described in this study might be partly explained by traditional risk factors and novel risk factors. The main contribution of this reduction is due to the effects on inflammation, followed by the effects on glucose metabolism, insulin resistance, blood pressure, HDL, VLDL metabolism, although a minor contribution has been reported on LDL size and particles. However, additional unevaluated factors might participate to this association [7].

Estruch et al. reported that the adherence to Mediterranean diet might improve anti-inflammatory profile at a circulating and cellular levels; another study by Esposito showed that, after two-year follow-up of exposure to Mediterranean diet, this dietary pattern was associated with an improvement in hsCRP concentrations and endothelial function. In the light of these findings, the attenuation of the inflammatory processes represents a mechanism mediating some of the beneficial effects of Mediterranean dietetic regimen on cardiovascular diseases.

#### 3.4.1. Role of Oxidative Stress

In the pathogenesis of cardiovascular diseases, oxidative stress plays a central role, with the consequent formation of atherosclerotic plaques due to the increase in concentrations of oxidized LDL. The low intake of anti-oxidants in the diet can indirectly increase the risk of acute heart disease in apparently healthy people. The intake of β-carotene, vitamin C, vitamin E, natural folates, flavonoids, and minerals (such as selenium), included in a Mediterranean diet regime. seems to contribute to the reduction of this risk [184].

A significant reduction in circulating oxidized LDL and inflammation markers was achieved by implementing a Mediterranean diet, as shown by a new randomized clinical trial [185,186].

The oxidative stress and the inflammation are both crucial points in the pathogenesis of endothelial dysfunction, which contributes to the pathogenesis of atherosclerosis. Omega 3 fatty acids exert their anti-inflammatory effect through binding to the 120 G protein-coupled receptor and inhibition of inflammation NLRP3 [187,188]. Some phytochemicals contained in whole grains and in extra virgin olive oil have an anti-inflammatory action. A lot of phytoprotectants, such us ferulic acid, lignans, and phytic acid, are contained in aleuron layer of wheat bran, and they have antioxidative and anti-inflammation properties [189,190].

The whole grain contains a spermidine (polyamine) that seems to extend the life of human cells [191]. Histone acetyltransferases is inhibited by this polyamine and this causes a reduction in inflammation, cellular necrosis processes typical of aging and, finally, a greater resistance to oxidative stress.

Antioxidants compounds are richly represented in Mediterranean foods and seem to mediate the major beneficial effects of the Mediterranean diet, other salient mechanisms through which the Mediterranean diet exerts its central role in the context of the cardiovascular prevention are anti-inflammatory effects, modification of the microbiota, the modulation of gene, and protein expression [3]. Recently, a study by Kouli GM et al. [192] has showed that hydroxytyrosol exerts mechanism of repair on oxidative damage and determines an improvement of circulating lipids. This molecule is contained in fruits, nuts, legumes, and extra olive oil.

Some researchers observed a reduction of the circulating 8-hydroxy-2′deoxyguanosine (8-OHdG) levels in individuals on Mediterranean diet when compared to those on SFAs rich diet; this study by Urquiaga did not permit to define the exact contribute of a moderate intake of red wine because both the diets include it [193].

8-OHdG is an oxidized nucleoside of DNA. It is frequently detected in nuclear and mitochondrial DNA; as result of DNA repair, 8-OHdG is excreted in the urine. Numerous findings have reported that urinary 8-OHdG not only is a biomarker of generalized, cellular oxidative stress, but it could also be considered a risk factor for atherosclerosis, diabetes, and cancer. Some human studies described increased amounts of oxidatively modified DNA and 8-OHdG in human atherosclerotic plaques. Elevated urinary 8-OHdG were also detected in diabetic patients with hyperglycaemia; moreover, it has been observed that its urinary concentrations in diabetes correlated with the severity of diabetic complications (nephropathy, retinopathy) [194].

#### 3.4.2. Effects of Mediterranean Diet on Foam Cells Formation

Florez et al. conducted a study that showed how the extra virgin olive oil rich in linoleic acid induced a greater internalization of triacylglycerol in THP-1 macrophages, when compared to extra virgin olive oil rich in oleic acid [195]. The results that were obtained from this study evidenced that the different composition of fatty acids of extra virgin olive oil played an important role in the formation of foam cells. Indeed, studies that were conducted on THP-1 macrophages showed that fatty acids that are rich in oleic acid reduced the accumulation of lipids in macrophage cells. This would seem to be due to a down-regulation of apolipoprotein B48 receptor [196]. On the other hand, linoleic acid would seem to stimulate the CD36 receptor, leading to an accumulation of triacylglycerol in the cells [197].

CD36 is an important macrophage scavenger receptor that binds oxidized LDL [198]. Up-regulation of the CD36 receptor appears to be due to both increased stimulation of transcriptional and post-transcriptional mechanisms. Furthermore, it seems that PPARs play a role in this over-regulation by promoting the transcription of this receptor, since fatty acids are ligands of PPARα and PPARγ [199,200]. Accordingly, the expression of this receptor seems to play a role in the formation of foam cells and fatty acids would determine the regulation of gene expression [197].

### 3.5. Mediterranean Diet and Arterial Stiffness

Arterial stiffness is the ability of arteries to expand and contract in relation to the various stages of the cardiac revolution, converting intermittent blood flow into a more stable flow, and it can be considered as a potential factor in the beginning and progression of atherosclerosis as well as hypertension.

Therefore, increased arterial stiffness could serve as an “early marker” for discovering initial signs of atherosclerosis and/or arterial structural changes that are caused by various risk factors, including hypertension.

Epidemiological longitudinal studies showed an independent predictive value of arterial stiffness, the carotid pulse pressure, and the Augmentetion index (AIx), measured directly by a tonometer, for cardiovascular events [201].

Most of the evidence was given for aortic stiffness, as measured through the carotid-femoral pulse wave velocity (PWV).

In recent years, aortic stiffness has shown an independent predictive value for all causes of cardiovascular mortality and mortality, fatal and non-fatal coronary events, and fatal strokes in patients with uncomplicated high blood pressure, type 2 mellitus diabetes, and complicated kidney alterations [202,203].

In the last few years, many studies evidenced a straight interaction between a dietary pattern rich in vegetables (excluding potatoes), fruits, whole grains, nuts, legumes, fish, ratio of monounsaturated-to-saturated fat, all components of the typical Mediterranean Diet, was associated with a reduction in cardiovascular risk factor, and among these, even the arterial stiffness.

Van de Laar et al., in a longitudinal study of 373 patients observed between the ages of 13 to 36 years, tried to investigate whether adherence to a Mediterranean dietary pattern during adolescence and early adulthood affects arterial stiffness in adulthood. At the age of 36, individuals with higher carotid arteries stiffness had a less adherence to the Mediterranean Diet, defined by lower aMED scores, underling the importance of a correct alimentation, especially in the early stages of growth [204].

Rodriguez Martin et al., in a sub-analysis of the EVIDENT study consisting of 1553 subjects (aged 20–80 years) with no cardiovascular disease, evaluated the relationship between the EVIDENT diet index (0–100), good predictor of adherence to Mediterranean Diet, and cardiovascular risk and pulse wave velocity (PWV) of the sample. In the logistic regression analysis, adjusted for age, gender, smoking, energy intake, antihypertensive, antidiabetic and lipid-lowering drugs, and systolic blood pressure, it was shown that the increasing of the EVIDENT diet index was associated with a reduction of the PWV and, consequently, with a reduction of the cardiovascular risk emphasizing the importance of arterial stiffness as a predictor of CV event [205].

## 4. Mediterranean Diet and Coronary Artery Disease

Over the past decades, several studies reported that a strict adherence to Mediterranean diet might prevent inflammation, which exerts a central role in the pathogenesis of atherosclerosis and cardiovascular disease [69,206]. Further recent studies reported that better compliance with the Mediterranean dietary pattern might, over time, down-regulate the development of atherosclerosis in the coronary arteries [207,208].

A recent Turkish study reported the existence of a relationship between the extensiveness of angiographic quantitative coronary artery disease (CAD) adherence to Mediterranean diet [209]. These findings suggest that a higher compliance with the MD leads to lower extensiveness of CAD. To date, there are not sufficient data on highly probable association between Mediterranean diet and severity of CAD, although this eating pattern has been widely studied in the context of cardiovascular disease.

A recent study analysed the relationship between dietary patterns and a score evaluating coronary morphology, the SYNTAX score, and in coronary artery disease (CAD) patients from the INTERCATH study [210]. At logistic regression analysis, the strongest correlation with Mediterranean dietary pattern was found for hs-RCP and this association remained significant after adjustment for cardiovascular risk factors. These results suggest an independent association of adherence to Mediterranean diet with less complex CAD. Moreover, in the light of these results, Hs-CRP might appear as a marker of the vasoprotective effects of the Mediterranean diet. These results strengthen the evidence for the protective effect of a MeDi pattern in cardiovascular prevention.

Acar et al. evaluated patients who had a history of coronary revascularization at least six months before to enrolment [211]. An alternate MeDi score was calculated to evaluate the Mediterranean style dietary adherence. By applying this measurement, after a multivariate analysis the authors concluded that the adherence rate to a healthy diet was low in patients with previous coronary revascularisation. The Seven Countries Study was the first to underline the differences in CAD mortality rates between the 16-country cohort from northern to southern countries and also from Japan to the peculiar dietary patterns of each country of the participants and, in particular, it showed the important role of the intake of saturated fatty acids and flavonoids.

Investigators from the Lyon Heart Study [212] studied 605 patients aged 55–80 years with previous CAD; the authors observed that a better cardiovascular risk profile of the Mediterranean diet also extended to the clinical setting of the secondary prevention of cardiovascular disease. This study showed that patients that strictly followed a Mediterranean diet style had 50–70% lower risk of recurrent clinical events related to coronary artery disease.

These findings corroborate the role of the adherence to a Mediterranean dietary pattern as compared with other diet styles, in the context of a so-called prevention Step–I (i.e., total fat <30% of total calories, carbohydrates >55% of total calories, protein about 15% of total calories, and cholesterol <300 mg/dL).

The CARDIO2000 investigators [213] conducted a study in which they analysed 848 middle-aged and older patients with previous myocardial infarction and unstable angina and 1078 age- and sex- matched controls from Greece. They reported that a higher degree of adherence to a Mediterranean diet was linked to a significant rate reduction (−23%) of the risk of developing a first ischemic cardiac event and this coronary benefit was more significant in people who were living in rural areas as compared with subjects living in urban or semi-rural areas.

The same scientific group also reported the synergistic effect of the combination of the Mediterranean diet with statin treatment on coronary risk; in fact, they showed that the combination approach of diet + statin was associated with a 43% reduction in coronary risk, independently from cholesterol levels and other cardiovascular risk factors.

Recently, the PREDIMED study, a prospective study involving 22,043 adults from Greece [118], reported that a 20% increase in the Mediterranean diet score was associated with a 33% reduction in coronary heart disease mortality, thus highlighting the existence of a significant negative relationship between a high adherence to the Mediterranean diet and death from coronary heart disease.

The results of this study were irrespective of sex, smoking status, level of education, BMI, and physical activity, but strictly dependent from the age of patients with higher effects in patients ≥ 55 years, but not in those ≤ 55 years.

In the light of these findings, this study explains that a cultural adherence to Mediterranean diet, just the simple recent adoption of a healthy dietary pattern, seems to be the only practical way of heart protection.

What of the single elements of the Mediterranean diet have been reported to more strictly linked to cardiovascular risk reduction is an unanswered question. The “Habits in Later Life Only” study reported that only legumes intake was associated with a reduction in mortality hazard ratio, after adjustment for location/ethnicity of the analysed elderly people from Japan, Sweden, Australia, and Greece. Nevertheless, Dontas et al. [173] found that higher adherence to the Mediterranean diet was associated with 21% lower odds of being affected by one additional risk factor (i.e., hypertension, hypercholesterolemia, diabetes, and obesity) in women and with 14% lower odds in men, irrespective of various potential confounders. This observation could be directly related to the reduction of cardiovascular risk, and therefore to life extension.

## 5. Mediterranean Diet and Congestive Heart Failure

Heart failure (HF) is defined as a clinical syndrome that is secondary to the inability of the heart to adapt the cardiac output to the metabolic needs of the organism or, in any case, to be able to do so only at the expense of increasing the filling pressures in one or more chambers cardiac and venous circulation upstream.

According to more recent epidemiological data, the prevalence of HF has increased in recent years, by 1–2% in the adult population and by more than 10% in older subjects. The main risk factors are hypertension, obesity, type 2 diabetes mellitus, and, in general, acute cardiovascular diseases (CAD) [214]. The primary prevention for HF is currently one of the main public health battles. In recent years, important evidence has emphasized as lifestyle changes, which include changes in the individual dietary approach and play an important role in primary prevention for CVD [215].

As shown by the results of the PREDIMED Study, the Mediterranean diet (MD) is considered to be an effective strategy for the primary prevention for the development of cardiovascular disease (CVD), such as myocardial infarction and stroke [97].

Although this study was the first to suggest that Traditional Mediterranean diet (TMD) could have benefits on the primary prevention of heart failure (HF), which is justified by the data obtained, the effects on HF incidence in subjects who had adopted that dietary model was only a protocol-specified secondary outcome of PREDIMED.

To our knowledge, the beneficial effects of effective dietary patterns, which outsource the consumption of specific nutrients, on the primary prevention of HF have not been assessed by systematic review or meta-analysis. For this reason, D’Almeida et al., in a recent review in 2018, analysed five randomized controlled trials, seven court studies, and two cross-sectional studies, for a total of 188470 subjects, with the aim of defining the effects of six different dietary models on the primary prevention of HF [215]. Among the dietary regimens that have been studied by these authors, there are the Mediterranean diet (MD) and Dietary Approach to Stop Hypertension (DASH); the DASH diet is one of the recommended dietary models, because it can influence blood pressure, which is positively correlated with functional changes in the cardiac and endothelial levels [216,217]. D’Almeida et al. have observed that HF could be prevented by adhering to one of these two types of dietary patterns, although these data should be further studied and re-evaluated, according to the poor power of the findings.

In the specific case of the MD, the authors included four randomized controlled trials and four court studies in this systematic review. During the research of the various studies, one of the trials that was identified and subsequently inserted aimed to analyse the incidence of HF. Papadaky et al. have conducted a recent secondary analysis of the pre-specified outcome of the PREDIMED trial with the aim of assessing whether TMD could influence the incidence of HF [218]. This study included 7403 subjects with high cardiovascular risk divided in 3 groups with different dietary plans, with type 2 diabetes mellitus and/or at least 3 other CVD risk factors including family history of premature coronary heart disease, smoking, hypertension, low HDL-cholesterol levels, high LDL-cholesterol levels, or obesity (body mass index ≥25 kg/m^2^). This study, based on 7403 subjects with high cardiovascular risk and divided into three groups with different dietary models (TDM + EVOO, TMD + nuts and low-fat control diet), showed no significant association between adoption of TDM enriched with EVOO or nuts and no reduction of the incidence of HF. According to the observation of only 94 HF cases during the trial, the authors hypothesized that the results of this secondary analysis of a pre-specified outcome of the PREDIMED study were inconclusive due to the low power of the trial, in combination with evidence that dietary patterns might influence HF development differently, depending on the type, severity, and general etiopathogenesis of the syndrome [69,219].

The Mediterranean diet could partly change the incidence of HF due to the now known anti-oxidative and anti-inflammatory properties that characterize most of the components of this dietary model [220]. Several studies have shown that factors, such as oxidative stress [221] and inflammation [222], play an important role in the pathogenesis of HF.

Fito et al. in 2014 conducted a sub-sample study of 930 subjects at high cardiovascular risk previously enrolled in the PREDIMED study to show how adhesion to TDM could reduce the levels of important biomarkers of HF with special attention to *N*-amino terminal fragment of the prohormone BNP (NT-proBNP) [223]. These participants, who had not yet been diagnosed with CVD at the time of enrolment, were randomly assigned to one of the three dietary intervention groups. The results of this study showed that TMD lowers NT-proBNP levels as compared to the controlled diet, after one year of intervention. Another important finding was a reduction in concentration of oxLDL and lp(a) in participants that were assigned to the TMD + EVOO group. Therefore, the authors confirmed their hypothesis of a protective effect of TMD by promoting changes in HF biomarker values.

Brain natriuretic peptide (BNP) is a hormone that is encoded by a gene whose transcription is mainly induced by the stretching of the left ventricle wall in response to an increase in tensive values or volume. The proBNP, which is a peptide of 108 amino acids, is subsequently cleared into BNP, a peptide of 32 amino acids, and NT-proBNP, a peptide of 76 amino acids, biologically inert. NT-proBNP has recently been defined as a sensitive marker and predictive factor of acute HF [224], with an important prognostic meaning in this context [225].

Therefore, although cardiac wall stress is the main event that is responsible for releasing these molecules, a wide range of factors and structural and functional abnormalities of the heart can trigger an increase in BNP or NT-proBNP. In this context, among the factors that might influence the values of these two peptides, there are counted interluchin 1β (IL-1β) and another important pro-inflammatory cytokine as tumour necrosis factorα (TNFα), promoting the expression of the gene consideration [226]; the plasma concentration of TNFa and the expression of the IL1b gene have been associated with the consumption of TMD [227,228].

These observations were confirmed by data that were obtained by Fito et al., who showed a correlation between TDM and reduction of oxidative stress damage and TNF receptor expression (TNFR), in agreement with the study of Urpi-Sarda et al. [227], which observed how those who improved their diet towards a model of MD, in particular the consumption of its specific components, such as VOO and vegetables, reduced the conception of TNFR.

In recent years, an important role in promoting the development and progression of HF has been attributed to oxidative stress, an expression that indicates an excessive increase in the relationship between production and growth, by special systems, reactive oxygen species [229,230]. Mallat et al., observed increased levels of lipid peroxidase and 8-iso-prostaglandin F2alfa in patients with HF, which are important markers dosed in blood and pericardial fluid; these data are in accordance with a systematic review, published in the year 2011, which showed how optimizing ROS production could take a more important role in promoting oxidative stress in HF than reducing the effectiveness of antioxidant systems [221]. Mitochondria, NAD(P)H oxidase, xanthine oxidase are, together with nitric oxide synthetase (NOS), the main spring of ROS within myocardiocytes. Within the framework of these systems, special attention should be given to mitochondria, which physiologically produce low amounts of ROS during cellular respiration; mitochondrial dysfunction is therefore significantly associated with increased production of ROS, promoting oxidative stress [231]. The alteration of the balance between the production and inactivation of ROS, which take place through various mechanisms that include alterations to mitochondrial DNA (mtDNA), induces significant damage to mitochondria that results in dysfunction and activation of a vicious circle, characterized by further production of ROS and worsening of the mitochondrial dysfunction, which culminates in cellular damage [232].

Finally, the persistence of oxidative stress promotes the progression of HF by encouraging cardiac remodelling; chronically elevated ROS levels activate several signal transduction pathways and transcription factors that are involved in cardiac hypertrophy [233], induce apoptosis [234], and activate metalloproteinase (MMPs), enzymes that are involved in tissue remodelling [235] and whose activity was positively associated with HF, probably as a result of the ROS increase [236].

In this context, previous studies have suggested that the protective effect of MD on cardiovascular risk is partly mediated by the anti-oxidative properties of important foods that characterize this dietary model [237]. To our knowledge, the studies that were conducted with the aim of defining how MD reduces oxidative stress are limited. For this reason, Dai et al., in a twin study that were conducted in 2008 and based on 297 twins (81 monozygotic and 57 dizygotic pairs of twins, nine monozygotes, and 12 dizygotes unpaired twin), studied the beneficial effects of MD on oxidative stress [220]. Establishing the relationship between reduced glutathione (GSH) and oxidized glutathione (GSSG) as a plasma surrogate for oxidative stress, the results of the present study suggested that the adoption of a Mediterranean dietary model correlated with the increase in the GSH/GSSG ratio, consistently with the data obtained from previous experimental studies on animals that have emphasized as typical components of MD foods, such as polyphenols, favoured a reduction of the oxidative stress defined by the decrease in the quantity of GSSG not associated with a change in the quantity of GSH [238,239]. The validity of these results stays precisely in the study design, paying particular attention to the choice of a sample of monozygotic and dizygotic twins belonging to the same family, which allowed for controlling genetic, behavioural, and socioeconomic factors, defined as the unmeasured confounding factors.

The MD can determine the increase of GSH/GSSG through different mechanisms. When considering that the GSSG, as obtained from the oxidation reaction of GSH catalysed by glutathione peroxidase [240], is again converted into GSH thanks to the simultaneous oxidation of NADPH [241], MD can partly reduce oxidative stress by increasing the availability of NADPH and the expression of glutathione reductase [242], the enzyme that catalyses the second of the two reactions described above. In this way, the decrease of GSSG, which results in an increase in the GSH/GSSG ratio, is mediated by the effects of nutrients and other components that are contained in the typical foods of the Mediterranean dietary pattern.

The increase in this ratio can be attributed to the reduced consumption of glutathione as the main antioxidant mechanism, since the MD is a dietary model that is enriched with other antioxidants as well known, such as vitamin C, vitamin E, carotenoids, polyphenols, zinc, and selenium [243].

Resveratrol is a type of natural phenol, a stilbenoid that is contained in blueberries, the skin of grapes, raspberries, and peanuts. Several studies have described its benefits. Yeung et al. analysed numerous research publications on resveratrol investigating its effects on health. This work reported and underlined the antioxidants activity, the antidiabetic, and antimicrobial properties of this phenol; moreover, it described the role of resveratrol as mediator of anti-proliferative and anti-inflammatory activities via gene expression alterations and metabolic pathway modulations [244].

In addiction it is potentially involved in the prevention and treatment of cardiovascular diseases, cancer, neurodegeneration, obesity, and diabetes.

Among the predictors of morbidity and mortality in chronic HF, there are low-density oxidized lipoprotein (oxLDL) plasma levels [245,246]. Fito et al. observed a reduction in oxLDL levels of approximately 8 U/L in subjects who followed an MD model for one year. In addition, in 2008, Bergmark et al. conducted a study that highlighted how oxidized phospholipids were transferred from oxidized LDL predominantly to lipoprotein a (Lp(a)), which is an LDL molecule that is linked to apolipoprotein (a) rich in carbohydrates (apo(a)) [247]. In the control group, an increase in Lp (a) plasma levels was obtained, which was attributed to the increase in other lipoprotein plasma markers rich in triacylglycerol (TRL) and in apolipoprotein (apo) CIII non-HDL [248]. In the TDM groups the same results were not recorded, and even the TMD + VOO promoted a significant reduction in the levels of LP (a) when compared with the control group. Fito et al. also found a reduction in TRLs after TMD adoption [249], which confirmed what was previously hypothesized.

## 6. Mediterranean Diet and Stroke

The Seven Countries Study shows how the Mediterranean diet, followed by the population of Crete, is the best in terms of prevention against stroke. This study reports that the coronary risk in Finland is 15 times greater than that in Crete, which is 40% of that in Japan [250].

The Mediterranean diet is described by Ancel Keys as ‘a mainly vegetarian die favouring fruit for dessert’. In fact, this diet provides for reduced glycemic intake with a fatty acid intake of 40%, represented mainly by healthy fatty acids, which are associated with a higher consumption of fruit and foods with a high fiber content.

In confirmation of this observation, a study that was conducted in India [251] showed how the Mediterranean diet that was practiced for two years, as compared with a diet poor in fat, resulted in a 50% reduction of coronary and cerebrovascular events, although 60% of subjects had a vegetarian diet at the time of enrolment.

A recent meta-analysis [252] indicated that an absolute adherence to the Mediterranean diet, independently from the population analysed, defined a lower risk of stroke both ischemic and haemorrhagic (about 16% for each four points higher in the MedDiet Score).

In fact, as shown by the results of the INTERSTROKE study, there is a considerable reduction in the risk of stroke related to the diet practiced, in all of the different ethnic groups that were studied [253].

A multicentric randomized study was conducted in Spain [183], in which the participants (7447 enrolled patients) with a high risk of developing cardiovascular diseases were divided into three arms: one that was given an indication to practice Mediterranean diet supplemented with extra virgin olive oil, one that was given an indication to practice Mediterranean diet supplemented with nuts, and, finally, a control group that was supposed to practice a hypo-caloric diet. The follow up was three-monthly and each participant received the amount of oil or nuts that he would have to consume. The number of major cardiovascular events represented the primary endpoint. These events occurred overall in 288 subjects of which 96 were part of the group that practiced a Mediterranean diet supplemented with extra virgin olive oil (3.8%), 83 were part of the group that practiced a Mediterranean diet supplemented with nuts (3.4%), and 109 belonged to the control group (4.4%). However the results obtained concerned the combined cardiovascular endpoint, represented by the risk of developing cardiovascular and cerebrovascular events. Data from the PREDIMED trial are in line with the results that were obtained from other observational studies, which have shown how the Mediterranean diet has protective effects in the cardiovascular field [254,255]. In fact, in the Three-City Study, it examined the correlation between the consumption of extra virgin olive oil/plasma concentration of oleic acid and the incidence of stroke. The follow up was five years. The results obtained, adjusted for socio-demographic, physical, and body mass index variables, and also for individual risk factors for stroke, showed an inverse correlation between the incidence of stroke and the consumption of extra virgin olive oil. Patients that consumed greater quantities of extra virgin olive oil had a risk of stroke of 41% lower, as compared with participants that have never consumed extra virgin olive oil.

According to the findings of several studies, the beneficial effects that were induced by the Mediterranean diet are determined by its action towards the main risk factors for stroke, such as inflammation, oxidative stress, endothelial dysfunction, platelet aggregation [256,257], and diabetes [258].

The reduced consumption of saturated fatty acids, as demonstrated by epidemiological and prospective studies, is associated with a reduction in plasma LDL cholesterol concentrations and a consequent reduction in the incidence of cardiovascular and cerebrovascular events. This happens especially when polyunsaturated and monounsaturated fatty acids are replaced with saturated fatty acids [259].

In confirm of this, in several randomized controlled clinical studies, it was shown that the intake of exclusively vegetable polyunsaturated fats has an effect in reducing the incidence of cardiovascular diseases equal to 30%, a result that is similar to that obtained through statin therapy [260].

In this regard, two studies, the Lyon Diet Heart Study [212] and the Scandinavian Simvastatin Survival Study (4Sstudy) [261], showed how the implementation of a Mediterranean dietary regime had protective effects from the cardiovascular and cerebrovascular accidents. In fact, the Lyon Diet Heart Study showed the incidence of coronary events and stroke was reduced by 70% in four years in patients that practiced a Mediterranean diet. In the 4S study, instead, the intake of simvastatin reduced recurrent coronary events by 40% in six years. It is important to note that patients enrolled in both studies already presented a major cardiovascular event in the past.

At the moment, no study has fully cleared in subjects with acute ischemic stroke the relationship between adherence to a MeDi style and the different diagnostic subtype or severity of stroke.

A retrospective study [262] analysed the association between MeDi adherence that was assessed by calculating Mediterranean Diet Score (MeDi score) and TOAST stroke subtype, severity index, and outcome of ischemic stroke. This study reported that stroke patients had a significantly lower mean MeDi score when compared to control subjects.

Authors reported a negative association between MeDi score and National Institutes of Health Stroke Scale (NIHSS) and Rankin scores, showing a negative relationship between adherence to a Mediterranean diet style and severity of ischemic stroke concerning acute neurological deficit at admission and disability at discharge.

The MeDi Score significantly varies in relation to the stroke subtype. In fact, the Large Artery Atherosclerosis stroke (LAAS) had a low-to-medium score as compared to the lacunar subtype and cardio-embolic subtype. This suggests an inverse relationship between adherence to the Mediterranean diet and atherothrombotic pathogenesis of ischemic stroke. The results that were obtained suggest that patients with a lower MeDi score are more susceptible to developing ischemic strokes. Although some plausible bio-molecular mechanisms underlying this correlation have been validated, it is necessary to carry out further studies that aimed at evaluating the different degrees of acute inflammation in the post-ictal phase in relation to the dietary regime practiced by the patient.

## 7. Conclusions

**Figure 3:**
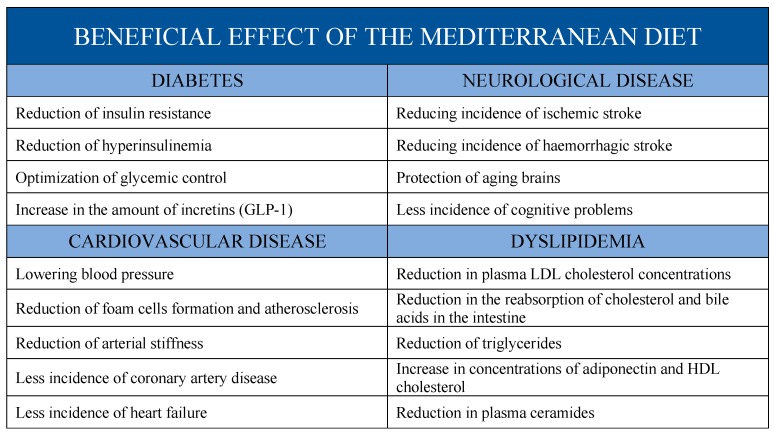
Beneficial effect of the Mediterranean diet.

Over the past few decades, there has gradually been an increase in awareness that dietary pattern affects health, in particular numerous studies have reported notable effects of nutritional components on metabolic functions of organisms and pathological processes (See Figure 3). Among several dietary pattern, the Mediterranean diet has been widely recognized as a model of “healthy eating”, because of its contribution to the state of health and its positive influence on quality of life. The traditional Mediterranean diet consists of a wide range of minimally processed whole grains and legumes, fresh vegetables, fresh fruits, cold pressed extra virgin olive oil, nuts, and seeds as the principal source of fat, moderate consumption of fish, low amounts of dairy products, very low frequency of intake of red and processed meat, and wine consumed in low to moderate amount.

Between 2006 and 2010, subsequent interesting studies highlighted the role of virgin olive oil in the modulation of endothelial function in the context of a Mediterranean dietary regimen. It is now known that the Mediterranean diet has undoubted health benefits compared to other dietary patterns, however some studies, after a careful reassessment, suggest that the Mediterranean diet is not free from defects and some researchers have exposed criticism and doubts. In the light of these reflections, the exponents of international institution, while taking the evolution of the Mediterranean diet into account, proposed a new food pyramid for the modern Mediterranean diet. The modern food pyramid has a lower base that emphasizes the importance of a daily adequate level of physical activity and it suggests a minimal consumption of alcohol and supplements of vitamins and minerals.

Animal and human translational studies have recently brought to light some biological processes that seem to mediate the beneficial effects of the Mediterranean diet. The Mediterranean diet is the most studied dietary pattern at the level of evidence-based medicine. The high intake of fiber rich energy poor plant foods, the reduced content of sulfur and branch chain amino acids, and saturated fatty acids represent some of the most important elements that mediated the positive and pro-longevity effects of this dietary pattern. The gut microbiome is emerging as a relevant key factor that influences metabolic and molecular health; these effects are mediated by processing of numerous plant foods packed with fiber, vitamins, and phytochemicals. Nutritional genomics include several aspects of nutrition related molecular processes, such as transcriptomics, genomics, metabolomics, and genetics

To date, the current metabolomic literature, which has already identified specific important metabolites that result from the ingestion of unique diets and that influence health status, has reported interesting but often inconclusive evidence about Mediterranean diet and microbioma relationship, however it appears clearly defined the role of gut microbiome in contributing to the beneficial healthy effects associated with the Mediterranean diet.

Further mechanisms are certainly involved, thus further researches are needed to clarify known mechanisms and understand other processes underlying the interactions among food and nutrients, intake, single nutrient changes, microbiota, physical exercise, genetic predisposition, and their effects in modulating specific molecular pathway responsible of cellular, tissue, and organ function during aging [263,264,265,266,267].

The knowledge of gene-diet interaction is actually limited and still evolving. The studies on epigenome changes are helping to clarify and understand diversities in individual susceptibility, thus this research also aims to develop epigenomic drugs and look forward foods and supplements that will modulate epigenome.

In the light of these premises, it is desirable to promote scientific researches regarding genetic and biomolecular mechanisms that, besides giving a better explanation of the cause-effect relation between adherence to Mediterranean diet and reduction of cardiovascular diseases, it will provide the opportunity of personalizing the dietary regimen of a specific subject while also taking into account his genetic susceptibility.

## Figures and Tables

**Table 1:** Main studies about Mediterranean diet and its effects on diabetes.

Author and Year	Brief Description	Conclusions
Due, A. et al. 2008 [81]	46 nondiabetic, obese men (20) and premenopausal women (26) randomly assigned to 1 of 3 diets: (1) MUFA diet (*n* = 16): moderate in fat (35–45% of energy) and high in monounsaturated fatty acids (>20% of energy); (2) LF diet (*n* = 18): low-fat diet (20–30% of energy) (3) control diet (*n* = 12)	A diet high in monounsaturated fat has a more favourable effect on glucose homeostasis than does the typical Western diet in the short term and may also be more beneficial than the official recommended low-fat diet during a period of weight regain subsequent to weight loss.
Paniagua, J.A. et al. 2007 [82]	A prospective study performed in eleven (7 W, 4M) offspring of obese and type 2 diabetes patients randomly divided into three groups and underwent three dietary periods each of 28 days in a crossover design: (a) diet high in saturated fat (SAT) (b) diet rich in monounsaturated fat (MUFA; Mediterranean diet) (c) diet rich in carbohydrate (CHO)	Weight maintenance with a MUFA- rich diet improves HOMA-ir and fasting proinsulin levels in insulin- resistant subjects. Ingestion of a virgin olive oil-based breakfast decreased postprandial glucose and insulin conc entrations, and increased HDL-C and GLP-1 concentrations as compared with CHO-rich diet
Shah, M. et al. 2007 [91]	Test meals rich in palmitic acid, linoleic acid, oleic acid, and eicosapentaenoic acid (EPA) and docosahexaenoic acid (DHA) and containing 1000 kcal each were administered in a randomized crossover design to 11 type 2 diabetic subjects	In comparison with palmitic acid and linoleic acid, oleic acid or EPA and DHA may modestly lower insulin response in patients with type 2 diabetes without deteriorating the glucose response. EPA and DHA may also reduce the triglyceride response
Perez-Jimenez, F. et al. 2001 [92]	Intervention dietary study with a saturated fat phase and two randomized-crossover dietary periods: a high-carbohydrate diet and a Mediterranean diet for 28 days each	Isocaloric substitution of carbohydrates and monounsaturated fatty acids for saturated fatty acids improved insulin sensitivity in vivo and in vitro, with an increase in glucose disposal. Both diets are an adequate alternatives for improving glucose metabolism in healthy young men and women.
Brehm, B.J. et al. 2009 [89]	Overweight/obese participants with type 2 diabetes (*n* = 124, age = 56.5 ± 0.8 years, BMI = 35.9 ± 0.3 kg/m^2^, and A1C = 7.3 ± 0.1%) were randomly assigned to 1 year of a high-MUFA or high-CHO diet	In individuals with type 2 diabetes, high-MUFA diets are an alternative to conventional lower-fat, high-CHO diets with comparable beneficial effects on body weight, body composition, cardiovascular risk factors, and glycemic control
Vessby, B. et al. 2001 [83]	The KANWU study included 162 healthy subjects chosen at random to receive a controlled, isoenergetic diet for 3 months containing either a high proportion of saturated (SAFA diet) or monounsaturated (MUFA diet) fatty acids. Within each group there was a second assignment at random to supplements with fish oil (3.6 g *n*-3 fatty acids/d) or placebo	A change of the proportions of dietary fatty acids, decreasing saturated fatty acid and increasing monounsaturated fatty acid, improves insulin sensitivity but has no effect on insulin secretion. A beneficial impact of the fat quality on insulin sensitivity is not seen in individuals with a high fat intake

**Table 2:** Main studies about mediterranean diet and its effects on hypertension.

Author and Journal	Brief Description	Conclusions
Toledo et al. 2013 [99]	The PREDIMED primary prevention trial is a randomized, single-blinded, controlled trial conducted in Spanish primary healthcare centers. 7447 men (aged 55 to 80 years) and women (aged 60 to 80 years) who had high risk for cardiovascular disease were assigned to a control group or to one of two Mediterranean diets. The control group received education on following a low-fat diet, while the groups on Mediterranean diets received nutritional education and also free foods; either extra virgin olive oil, or nuts.	Both the traditional Mediterranean diet and a low-fat diet exerted beneficial effects on BP and could be part of advice to patients for controlling BP. However, we found lower values of diastolic BP in the two groups promoting the Mediterranean diet with extra virgin olive oil or with nuts than in the control group.
Storniolo et al. 2017 [105]	Non-smoking women with moderate hypertension were submitted for 1 year to interventions promoting adherence to the TMD, one supplemented with extra virgin olive oil (EVOO) and the other with nuts versus a control low-fat diet (30 participants/group). BP, NO, ET-1 and related gene expression as well as oxidative stress biomarkers were measured.	The changes in NO and ET-1 as well as ET-1 receptors gene expression explain, at least partially, the effect of EVOO or nuts on lowering BP among hypertensive women.
Nissensohn et al. 2016 [98]	Six trials (more than 7000 individuals) were identified. Meta-analysis showed that interventions aiming at adopting an MD pattern for at least 1 year reduced both the systolic BP and diastolic BP levels in individuals with normal BP or mild hypertension.	A positive and significant association was found between the MD and BP in adults.
Moreno-Luna et al. 2012 [112]	Double-blind, randomized, crossover dietary- intervention study. After a run-in period of 4 months (baseline values), two diets were used, one with polyphenol-rich olive oil (∼30 mg/day), the other with polyphenol-free olive oil. Each dietary period lasted 2 months with a 4-week washout between diets	The consumption of a diet containing polyphenol-rich olive oil can decrease BP and improve endothelial function in young women with high-normal BP or stage 1 essential hypertension.
Alonso et al. 2004 [122]	Prospective cohort study whose members are all university graduates to assess the risk of hypertension associated with olive oil consumption.	In a Mediterranean population, we found olive oil consumption to be associated with a reduced risk of hypertension only among men.
Psaltopoulou et al. 2004 [117]	Arterial blood pressure and several sociodemographic, anthropometric, dietary, physical activity, and clinical variables were recorded at enrollment among participants in the Greek arm of the European Prospective Investigation into Cancer and Nutrition (EPIC) study.	Adherence to the Mediterranean diet is inversely associated with arterial blood pressure, even though a beneficial component of the Mediterranean diet score-cereal intake-is positively associated with arterial blood pressure. Olive oil intake, per se, is inversely associated with both systolic and diastolic blood pressure.
